# A Study on Coexistence Capability Evaluations of the Enhanced Channel Hopping Mechanism in WBANs

**DOI:** 10.3390/s17010151

**Published:** 2017-01-14

**Authors:** Zhongcheng Wei, Yongmei Sun, Yuefeng Ji

**Affiliations:** State Key Laboratory of Information Photonics and Optical Communications, Beijing University of Posts and Telecommunications (BUPT), No. 10 Xitucheng Road, Haidian District, Beijing 100876, China; godframe@bupt.edu.cn (Z.W.); jyf@bupt.edu.cn (Y.J.)

**Keywords:** WBANs, channel hopping mechanism, channel conflict, coexistence capability estimation

## Abstract

As an important coexistence technology, channel hopping can reduce the interference among Wireless Body Area Networks (WBANs). However, it simultaneously brings some issues, such as energy waste, long latency and communication interruptions, etc. In this paper, we propose an enhanced channel hopping mechanism that allows multiple WBANs coexisted in the same channel. In order to evaluate the coexistence performance, some critical metrics are designed to reflect the possibility of channel conflict. Furthermore, by taking the queuing and non-queuing behaviors into consideration, we present a set of analysis approaches to evaluate the coexistence capability. On the one hand, we present both service-dependent and service-independent analysis models to estimate the number of coexisting WBANs. On the other hand, based on the uniform distribution assumption and the additive property of Possion-stream, we put forward two approximate methods to compute the number of occupied channels. Extensive simulation results demonstrate that our estimation approaches can provide an effective solution for coexistence capability estimation. Moreover, the enhanced channel hopping mechanism can significantly improve the coexistence capability and support a larger arrival rate of WBANs.

## 1. Introduction

Biomedical sensors, motion sensors, and other wearable devices are usually deployed on, near, or in human bodies to monitor vital signals (e.g., ECG, EEG, EMG, blood oxygen, blood glucose, blood pressure and heart rate) or non-medical signals (e.g., GPS, acceleration and rotation). These wearable devices networking together forms Wireless Body Area Networks (WBANs), which are the most promising technologies in many rising and interesting applications, such as remote health care, sports, and entertainment [1,2,3,4,5]. However, coexistence of multiple WBANs is an inherent and severe challenge due to natural mobility of human beings [6]. When multiple WBANs come in the proximity of one another, they suffer from serious interference and collision problems [7,8].

Channel hopping is a trivial solution but substantially rewards coexistence resolution [9]. In IEEE 802.15.6, multiple channels (from 10 to 79) are specified in each communication frequency-band. Each WBAN periodically changes its communication channel according to a pre-defined hopping sequence so as to mitigate interferences or to avoid collisions with other coexisting WBANs. As specified in IEEE 802.15.6, Channel Hopping, Beacon Shifting, and Active Superframe Interleaving are three basic coexistence technologies, among which channel hopping is the most supportable for capacity and mobility because of its all narrow band frequencies applicability [10]. A WBAN switching to a free channel achieves a direct benefit by preventing interferences and collisions from other WBANs [11]. By prearranging channel switching information, a seamless communication between the coordinator and sensors could be provided [12].

However, channel hopping suffers from several issues, such as energy waste, long latency and communication interruptions in WBANs. It is more challenging for switching channels in WBANs than in traditional ad hoc networks, because WBAN nodes share the same mobility pattern with the human body and more communicating node-pairs are coupled together in each channel switching operation [13]. George Smart et al., proposed decentralized time synchronized channel swapping (DT-SCS) [14]. Quick convergence, high bandwidth utilization, and high connectivity are achieved by balancing the number of nodes in each channel and swapping peer-to-peer requests/acknowledgments between concurrent transmitters in neighboring channels. Only a couple of nodes need to switch the communication channel, which does not meet the requirement of WBANs with a group of nodes in the same channel. In 2L-MAC [15], the updated channel information is delivered to the sensor via the next polling frame, and the hub needs an ACK frame to confirm that the sensor has received the updated channel information. The channel information exchanging between the hub and sensors introduces additional communications overheads. In dense WBANs, the hub has may generate extra latency for receiving ACK frames from sensors. Both the energy consumption and latency can be reduced by abandoning ACK frames. However, once some sensors fail to receive the channel switching commands, the hub has to notify these sensors again by sending additional channel switching commands, which leads to terrible communication interruptions. In the worst case scenario, the communications interruptions may lead to the death of patients, who may try to send critical medical measurements or life saving actuators messages (such as the readings of glucose actuators and cardiac pacemakers) to the hub [16]. Therefore, the frequency of channel switching plays a significant role in the coexistence performance and minimizing the frequency of channel conflicts will provide a new avenue for coexistence of WBANs.

The minimization of channel conflict probability is challenged by limited channel resources. Only 10 channels are specified from 402 to 405 MHz band in IEEE 802.15.6. In some dense crowds (e.g., more than 10 persons in the same elevator), number of channels is very limited to enabling the communications among sensor nodes, which may result in channel conflict. Fortunately, multiple WBANs can also be allocated to the same channel by beacon shifting [17], active superframe interleaving [18] and many other traditional coexistence techniques, such as CSMA [19], TDMA [20], hybrid MAC [21,22], etc. CSMA has an inherent coexistence capability by exploiting Clear Channel Assessment (CCA) mechanism [19]. Two or more WBANs are enabled to share a common TDMA schedule rather than sending data independently [20]. However, when large numbers of nodes are located in the same vicinity and with large traffic, CSMA suffers from serious collisions and TDMA supplies a small number of WBANs with limited time slots. Nevertheless, these traditional coexistence techniques provide a huge opportunity for channel hopping to further reduce the channel conflict probability. In some hybrid coexistence techniques, channel hopping is considered as a backup mechanism, which is only applied when the original channel allocation methods are failed [11]. To best of our knowledge, there is no literature focusing on modifying channel hopping in order to improve its coexistence capability.

This paper extends the channel hopping mechanism to be more general. Multiple WBANs are allowed to be allocated to the same channel by employing other coexistence technologies. Because these technologies are all operating in single channel, we call them Channel-shared Coexistence Technologies. Different from channel hopping studies in traditional ad hoc networks [14] and our previous work in WBANs [23] (i.e., a collision analysis study in duty-cycled and CSMA-based WBANs), where links/nodes are independent individuals and could be regarded as “link/node level” works, this paper considers each WBAN as a basic unit, and thereby it could be regarded as a “WBAN level” work. Since we define Coexistence Capacity (CC) as the maximum number of WBANs that a channel can support, the coexistence capacity extension of the channels provides an opportunity to reduce the probability of channel conflicts. This paper treats the resources as logical blocks allocated to each WBAN, which decouples the implementation from integrating different channel-shared coexistence technologies. We adopt a bitmap, in which the number of columns is equal to the number of channels and the number of rows is equal to the value of CC. The coexistence behaviors are considered to be a Birth-and-Death Process with interfering WBANs arriving, staying, and leaving (including queuing and non-queuing behaviors). Without loss of generality, we assume that each WBAN is independent with each other. So the channel sequences of the coexisting WBANs are independent and identically uniform distributed variables.

This paper investigates the coexistence capability in the view of channel conflict probability estimation, which is a macroscopic analysis based on resource allocations and could be adopted by basic channel hopping mechanisms for the performance enhancement. The studies are also suitable for active superframe interleaving, TDMA, etc., and can be applied to many other resource block allocation methods. The contributions of this paper are described as follows:

1. An enhanced channel hopping mechanism is proposed. We extend the channel hopping techniques to be more general and multiple WBANs are allowed to be allocated to the same channel. With limited coexistence capacity extension, coexistence capability is substantially improved.
2. A comprehensive coexistence capability analysis construction is provided. The conflict possibility of coexisting WBANs is described by a risk parameter and the coexistence behavior is modeled as a birth-and-death process. Both queuing and non-queuing behaviors are taken into account in our analysis.
3. Two approximate methods are illustrated to estimate the coexistence capability of the enhanced channel hopping mechanism. Numerous simulation comparisons are implemented to evaluate the availability and precision of our methods.

The remainder of this paper is organized as follows: The coexistence technologies overview and detailed motivation of the integration between channel hopping and channel-shared coexistence technologies are presented in Section 2. The models assumption and problem statement are illustrated in Section 3. The enhanced channel hopping mechanism and analysis construction of coexistence capability are proposed in Section 4. Section 5 presents the detailed coexistence capability estimation models for the enhanced channel hopping mechanism, including queuing and non-queuing behaviors. Extensive simulation results are illustrated and discussed in Section 6. Section 7 concludes this paper. In addition, all abbreviations and symbols are illustrated in Appendixes Appendix A.1 and Appendix A.2, respectively.

## 2. Related Works

In this section, we firstly illustrate the coexistence technologies comprehensively, including IEEE 802.15.6 coexistence strategies and some other channel-shared coexistence technologies; Then we discuss the opportunity of channel hopping coexistence capability extension.

### 2.1. Coexistence Technologies Overview

#### 2.1.1. IEEE 802.15.6 Coexistence Strategies

Beacon shifting, channel hopping, and active superframe interleaving are three common techniques employed to solve coexistence problems [17]. Several WBANs can share the same channel by interleaving their active superframes. Command-Active Superframe Interleaving Request frame and Command-Active Superframe Interleaving Response frame are employed to exchange the acknowledgments of active and inactive periods among coexisting WBANs. The hub adjusts its beacon period (superframe) length and inactive duration to enable the offered active superframe interleaving before sending its response. In $B^{2}IRS$ [18], the hub keeps its radio on so as to detect the other coexisting WBANs. Once it receives a beacon from a neighboring WBAN, it reschedules the active period after the neighbor. Thus WBANs in the same channel would not overlap their active periods. However, active superframe interleaving provides coexistence resolution only for static WBANs in residential environment or hospital with single patient node and fixed bedside hub.

Beacon shifting and channel hopping provide mobility support for more WBANs applications. When beacon shifting is available, beacons will be transmitted at different time offsets relative to the start of the beacon periods. A beacon shifting sequence not used by its neighbor hubs is chosen to mitigate potential repeated beacon collisions and scheduled allocation conflicts between overlapping or adjacent WBANs operating in the same channel. Seungku Kim et al., presented a distributed TDMA-based interval shifting scheme to avoid the wakeup of each WBAN coinciding with other networks by employing carrier sensing before a beacon transmission [17]. A tradeoff between the delivery-latency and energy consumption has been achieved by adjusting the length of the carrier sensing period according to the application requirements. While beacon shifting suffers from terrible beacon collisions when large numbers of WBANs are in the same vicinity and supports only 8 WBANs at most.

Differed from beacon shifting, channel hopping supports more WBANs coexistence (at least 10 WBANs) and is available with all narrow band frequencies specified in IEEE 802.15.6, P.S., Beacon shifting is not applicable when given Listen Before Talk (LBT) restrictions at 402 to 405 MHz band. Muhammad Mahtab Alam et al., compared channel hopping approach with time shared scheme in their work [24]. They found channel hopping approach outperforms the time shared scheme in all the metrics (i.e., packet error ratio, average packet reception ratio, average packet delay and average energy consumption), especially at lower transmission power. Judhistir Mahapatro et al., [12] proposed an Interference-Aware Channel Switching algorithm (InterACS), where the coordinator periodically monitors the level of Signal-to-Interference Ratio (SIR) and when it finds the SIR of the device below a threshold value, it switches to a different channel. In isMAC [21], if the WBAN leader detects a collision, it calculates a new inter-WBAN channel number. After sending the collected data to the central node, the WBAN leader notifies its member nodes with an information packet. Then, the WBAN leader changes the inter-WBAN channel to prevent collision and energy consumption. 2L-MAC [15] employed a threshold $H_{switch}$ to decide whether the backoff delay expires. Once the backoff delay expired, both the hub and the sensors switch to another channel to acquire more channel bandwidth. The hub needs an ACK frame from the sensor node to confirm that the updated channel information has been received. Therefore, switching a group of nodes into another channel will introduce additional resource waste by exchanging channel switching information. Once the sensors miss the beacon from the hub, they may lose connectivity and need additional overhead to recover. It’s problematic that the misinterpretation between hub and part of sensors might lead to serious harmfulness to users. Judhistir Mahapatro, et al., provided a seamless communication by prearranging channel switching information with at most three time broadcast, which achieves a tradeoff between energy waste/latency and robustness [12]. However, when quantity of sensors are equipped, it is hard to confirm that each sensor has received the channel switching information successfully within their limited broadcast repetation (three times) and the reception of ACK frames from the sensors still wastes abundant energy and leads to additional latency.

Consequently, channel hopping brings substantial coexistence improvement for WBANs. However, the operation of channel hopping usually leads to resources consumption, long latency, communication interruptions, etc. The frequency of channel hopping plays a significant role in the performance of the coexisting WBANs and the channel conflict probability really needs a comprehensive estimation.

#### 2.1.2. Other Channel-Shared Coexistence Technologies

Besides beacon shifting and active superframe interleaving, there are numerous technologies focusing on multi-WBAN coexistence at the same channel, such as CSMA, TDMA, etc. As two common MAC protocols, CSMA and TDMA are usually employed to avoid collisions and mitigate interferences. CSMA has a natural coexistence capable (i.e., multiple WBANs are allowed to share the same channel if some collisions are tolerated) [23]. WBANs are also able to coexist if time slots are rescheduled to avoid repeated collisions [25]. Md. Asdaque Hussain et al., provided a directional MAC approach by employing multiple beam adaptive arrays (MBAA) at the WBAN coordinator node [26]. When used as a WBAN coordinator, MBAA can successfully receive two or more overlapping packets at the same time. But the coexistence capability of the above channel-shared coexistence technologies are limited. Particularly, when dense WBANs co-located in the same vicinity, the performance decreases rapidly.

Nevertheless, integrating channel-shared coexistence technology will provide an attractive motivation to exploit the coexistence capability of channel hopping mechanism, which will bring more realistic benefits on coexisting WBANs.

### 2.2. Channel Hopping Enhancement Opportunities

As described in Section 2.1, channel hopping is suffering from the frequency band resources limitation. As shown in Table 1, from 402 to 405 MHz band, only 10 channels are available. Meanwhile, IEEE 802.15.6 requires that the protocol supports at least 60 sensors in $6^{3}$ m${}^{3}$ space. In fact, there are more WBANs than the lower bound of density required, e.g., queuing area in the hospital, subway station, supermarket, elevator, etc. Traditional channel hopping investigation only focused on generating the channel hopping sequences or implementing the specific procedures of channel hopping mechanism. In IEEE 802.15.6, the maximum-length Galois Linear Feedback Shift Register (LFSR) is provided. The channel hopping sequence generator aims at preventing the second channel conflicts, but barely benefits the probability reduction of the first channel conflicts. Cho et al. [27] achieved a performance improvement by employing a 4-channel adaptive frequency hopping scheme using the 30–120 MHz band. However, none of them respected on improving the coexistence capability of channel hopping.

In some hybrid coexistence techniques, channel hopping is employed as an assisted mechanism [11]. IEEE 802.15.6 also provides coexistence applicability guides. A hub may employ one or more of these mechanisms based on a trade-off between simplicity and effectiveness, and between feasibility and difficulty, as well as on their applicability to the operating frequency bands as noted in Table 2. Three mobility levels, designated as static (S), semi-dynamic (SD), and dynamic (D), are referenced in the table. Some examples of mobility levels are given as follows:

Static (S)-a single WBAN in a residential environment or a hospital with a single patient node and a fixed bedside hub;

Semi-dynamic (SD)-slowly moving ambulatory patients in an elder care facility requiring infrequent and/or event-based low-rate data transfers;

Dynamic (D)-fast moving ambulatory patients in a hospital with a large number of WBANS collecting continuous data traffic from many sensor nodes.

Integrating channel hopping and other coexistence technologies provides a potential coexistence capability improvement. Therefore, it is meaningful to ascertain the maximum number of WBANs that a single channel can support. Buddhika de Silva et al., have provided the experiment of packet drop rate (PDR) performance versus the number of WBANs and transmission rate [7]. When the number of WBANs increases to 5, the PDR becomes too lower to satisfy the requirement, especially when the transmission rate is 100 pkt/s, the PDR decreases rapidly. They also studied the effect of sensors’ locations when they are deployed on the body and on the table, respectively. When the number of WBANs is increased to 4, the PDR according to the location “on the body” is below 90%. Shipeng Liang et al., set up a WBAN testbed to investigate the impact of inter-user interference on various performance metrics when different severity levels of interferences are presented in the beacon-enabled mode [28]. When more and more jammers (interfering node on the table) are activated, both throughput and beacon delivery ratio (BDR) decline consecutively. Meanwhile, for each successful packet, both the number of backoffs and the number of transmissions significantly increase from no jammer to 4 jammers. Furthermore, when 3 interfering WBANs are active, only 7 Kbps throughput and 20% BDR can be achieved, compared with near 30–40 Kbps throughput and at least 90% BDR achieved by no interfering WBAN scenario. In 2L-MAC [15], authors compared their protocol with Polling, they found their protocol can achieve a better data delivery rate and data delivery latency. However, when there are 7 WBANs and the data size reached to 6, they still suffer low data delivery rate below 0.8 and high data delivery latency upon 50 ms. Once the channel hopping mechanism is enabled, 2L-MAC can support up to 20 WBANs with data delivery rate more than 0.9 and with data size no longer than 10 Kb. In consequence, channel-shared coexistence technologies support at most 6 WBANs in general. In this paper, we investigate coexistence capability by setting channel capacity from 1 to 6.

As mentioned above, the frequency of channel hopping plays a significant role in the performance of the coexisting WBANs and how to design an enhanced channel hopping mechanism so as to exploit the coexistence capability effectively will bring more realistic benefits on coexisting WBANs. Still, there is no coexistence capability quantification work for channel hopping and estimating the probability of channel conflicts becomes a critical issue in coexisting WBANs.

## 3. Models Assumption and Problem Statement

### 3.1. Models Assumption

#### 3.1.1. Coexistence Structures and Coexistence Capacity

**Figure 1:**
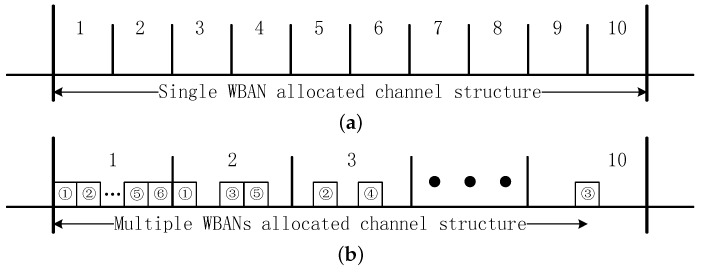
Channel structure illustration with the number of channels equaling 10. (**a**) 1-tolerated channel conflict model; (**b**) *m*-tolerated channel conflict model.

This paper investigates two types of channel structures for channel hopping mechanisms. Take 10 channels for instance, Figure 1a indicates the scenario that operations of different WBANs are not permitted in the same channel, which represents the implementation environment of plain channel hopping mechanisms. Once the coexisting WBANs are detected, the WBAN chooses another free channel and switches all their sensors to achieve an accessible operation [12]. We illustrate this structure to be single WBAN allocated channel structure. In Figure 1b, multiple WBANs are allowed to be allocated to the same channel, i.e., channel-shared coexistence technologies are integrated into channel hopping mechanism [11]. We illustrate this structure to be multiple WBANs allocated channel structure. Without loss of generality, this paper assumes that all the channels are identical. We employ a definition of coexistence capacity as the maximum number of WBANs that each channel supports. Thus the coexisting scenario under single WBAN allocated channel structure can be signed as 1-tolerated channel conflict model and the coexisting scenario under multiple WBANs allocated channel structure with *CC = m* can be signed as *m*-tolerated channel conflict model. As analyzed in Section 2.2, we investigate the channel coexistence capacity within the interval [1,6], i.e., *m* = 1, 2, 3, 4, 5, 6. Coexistence capacity provides a normalized formulation for channel hopping mechanisms, which benefits the coexistence capability analysis of these common channel hopping mechanisms.

#### 3.1.2. Channel Hopping Regulation

Channel hopping mechanisms are usually taking place when WBANs detect a coexistence conflict. There are many different channel hopping regulations, e.g., choosing a new channel randomly or orderly, centralized or distributed implementation, event or time driven, etc. As mentioned in Section 1 and Section 2, even channel hopping mechanism benefits coexistence performance, the consumption of channel hop is inevitable in coexisting WBANs, which are additional shortcomings introduced by channel switching. Therefore, this paper does not respect the specific mechanism implementation but focuses on the possibility of channel conflict when an interfering WBAN joins in the coexisting WBANs. Because of the human beings’ mobility and individualism, we assume that each interfering WBAN chooses the channel sequence randomly and the indexes of channel employed by different WBANs are independent with each other. Even the channel conflict occurs, the following channel sequence the interfering WBAN employs is still stochastic. Therefore, this paper chooses a normal channel assignment regulation that the sequences of different WBANs are following independent identical distribution.

#### 3.1.3. Stochastic Process Distribution and Queuing Model

**Figure 2:**
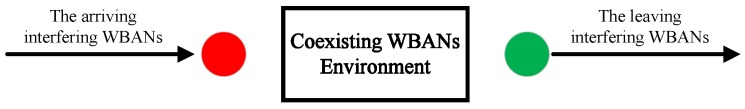
Coexisting WBANs system illustration.

**Figure 3:**
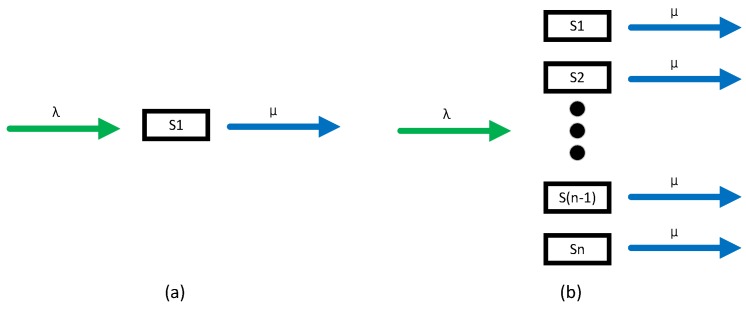
Illustrations of coexistence models. (**a**) Service-dependent; (**b**) Service-independent.

This paper considers coexistence behavior as a birth-and-death process, as shown in Figure 2. We suppose that the interfering WBANs stream is following Possion distribution with arriving rate *λ*, and the service time is assumed to follow negative exponential distribution. Two classical behaviors (i.e., humans queue up or not to take service) are taken into account in our coexistence analysis. For the first one, we suppose the coexisting WBANs environment to be one supplier of service, e.g., visiting a famous signature, buying tickets in a lobby, etc., in which customers queue up for service on first-come-first-served (FCFS) basis. We call this type of system Service-dependent coexisting WBANs, as shown in Figure 3a. For the second coexistence behavior, we suppose the coexisting WBANs environment to be multiple suppliers of service, e.g., dinning in a restaurant, shopping in a market, random walk across a square etc., in which customers need not queuing but take service directly. We call this type of system Service-independent coexisting WBANs, as shown in Figure 3b. For more queuing theory knowledge, please refer to [29,30,31].

### 3.2. Problem Statement

Channel hopping is an important coexistence technology, but brings serious shortcomings. Extension of channel coexistence capacity makes it a paradigm for coexistence resolution in WBANs. Integrating channel hopping and other coexistence techniques achieves obvious frequency reduction of channel switching and provides a new avenue in coexisting WBANs.

This paper provides an enhanced channel hopping mechanism, which aims at extending the application of channel hopping technique from 1-tolerated channel conflict model to *m*-tolerated channel conflict model. There is no doubt that enhanced channel hopping mechanism complicates the channel conflict analysis. In 1-tolerated channel conflict model, the number of busy channels is equivalent to the number of WBANs, and classical M/M/1 and M/M/c queuing models are suitable as basics for estimating the number of coexisting WBANs; while, for the *m*-tolerated channel conflict model, because the interfering WBAN joins in each channel randomly, the number of coexisting WBANs does not equal the number of busy channels any more, which fails the estimation methods based on M/M/1 or M/M/c queuing models.

## 4. Mechanism Description and Analysis Construction

This section presents the detailed description of enhanced channel hopping mechanism and provides the analysis construction of coexistence capability. As shown in Figure 2, the coexistence of WBANs can be considered as a system of coexisting WBANs environment running along with interfering WBANs arriving, staying and leaving.

### 4.1. Enhanced Channel Hopping Mechanism

The joining process pseudocode of enhanced channel hopping mechanism is shown in Algorithm 1. We take vector $\bar{X}$ =$\{\chi_{1},\chi_{2},\ldots ,\chi_{i},\ldots ,\chi_{n}\},0\leq i\leq n$ to record the statistics of channel occupied bitmap, in which $\chi_{i}$ denotes the number of WBANs coexisting in *i*-th channel, *n* denotes the number of channels. Let *m* be the coexistence capacity, and let *ι* be the channel index of the next interfering WBAN. Once the interfering WBAN detects that the *ι*-th channel is conflicted, a channel hopping sequence generator signed as $G(\iota ,m,\bar{X})$ will be employed to randomly get a free channel index. Since this paper does not respect the detailed method for generating the channel hopping sequence, to simplify our analysis, this paper assumes the generated sequence is following uniform distribution. Besides the joining process, enhanced channel hopping mechanism also provides $\bar{X}$ maintenance when interfering WBAN leaves. We omit the leaving process pseudocode.

**Algorithm 1** Joining process of enhanced channel hopping mechanism**Input**: Interfering WBAN channel index *ι*, coexistence capacity *m*, number of channels *n*, occupied channels map $\bar{X}$ and the channel hopping sequence generation $G(\iota ,m,\bar{X})$ **Output**: occupied channels map $\bar{X}$
1:**if** $Sum(\bar{X})\leq m\cdot n$ **then**2: **if** $\chi_{\iota }<m$ **then**3:  *join in the coexisting WBANs*4: **else**5:  *j← invoke $G(\iota ,m,\bar{X})$*;6:  *hop to the j-th channel*;7: **end** **if**8:**else**9: *refuse to join in the coexisting WBANs*;10:**end** **if**11:update $\bar{X}$


### 4.2. Analysis Construction of Coexistence Capability

The analysis construction of coexistence capability is composed of parameters and metrics which reflects the features and performances of coexisting WBANs system, respectively. Firstly, we provide a parameter to illustrate the channel conflict possibility when an interfering WBANs walks towards the coexisting WBANs environment, refer to Definition 1.

**Definition** **1.** For coexisting WBANs system with n channels specified, if κ channels are occupied/busy (i.e., n-κ channels are available/free), meanwhile the channel indexes of interfering WBANs are independent identically distributed, the channel conflict probability between the arriving interfering WBAN and coexisting WBANs is κ/n. Once the channel conflict occurs, the following channel hopping operation for interfering WBAN is inevitable. The channel conflict probability here is said to be Risk Parameter. On the contrary, (n-κ)/n is called Safety Parameter, which means that the interfering WBAN does not need to switch its channel and just joins the existing WBANs directly.

**Figure 4:**
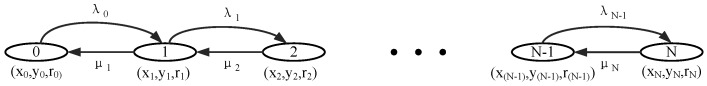
General analysis model for enhanced channel hopping mechanism in coexisting WBANs system.

To build a general analysis model, we set S={0,1,…,N}, which is the coexisting state space, *N* is the maximum index of coexisting state. Let $E=\{\left(x_{k},y_{k},r_{k}\right)/x_{k}\in X,y_{k}\in Y,r_{k}\in R,k\in S\}$ be the state parameter pairs, in which $X=\{x_{1},x_{2},\ldots ,x_{N}\}$ is called Coexisting-WBAN Parameter, indicating the number of coexisting WBANs, $Y=\{y_{1},y_{2},\ldots ,y_{N}\}$ is called Busy-channel Parameter, indicating the number of busy channels. Let $R=\{r_{1},r_{2},\ldots ,r_{N}\}$ be the Risk Parameter, which is depending on the ratio of busy channels according to Definition 1. As the coexisting WBANs system can be considered as a queuing system, the state transition diagram is illustrated as Figure 4.

Here, the reason why we do not employ *X* as the coexisting state space directly is to reserve the scalability for more complex queuing. For instance, the arriving customer sometimes is not single but group of WBANs and the Coexisting-WBAN Parameters here are not consecutive integers any more. As described in Section 3.1.3, this paper focuses on the general queuing model with arriving stream following Possion distribution and with service time following negative exponential distribution. Thus the value of $x_{k}$ in *X* is equal to *k* in *S*. Comparing with Risk Parameter which reflects the instantaneous conflict probability of interfering WBAN, the properties of coexisting WBANs system are more significant. Sign $p_{i}$ to be the probability of *i*-th state in steady state. The equilibrium equations are

(1) $$\left\{\begin{array}{c} \lambda_{0}p_{0}=\mu_{1}p_{1}\\ -\left(\lambda_{k}+\mu_{k}\right)p_{k}+\lambda_{k-1}p_{k-1}+\mu_{k+1}p_{k+1}=0\begin{array}{ccc} & & \left(k=1,2,\ldots ,N-1\right) \end{array}\\ \lambda_{N-1}p_{N-1}=\mu_{N}p_{N} \end{array}\right.$$

According to the probabilistic characteristics, we get Equation (2).

(2) $$p_{1}+p_{2}+\ldots +p_{N}=1$$

From Equations (1) and (2), we can get the probability distribution of *S*. Accordingly, we define the duration of each state as State Period and define the probability of each state as State Rate.

**Definition** **2.** For coexisting WBANs system, the probability of each state in Figure 4 is called State Rate. Accordingly, we define the Coexisting-WBAN Parameter Rate and Busy-channel Parameter Rate as the probability of Coexisting-WBAN Parameter and the probability of Busy-channel Parameter, respectively. Similarly, the duration of each state in Figure 4 is called State Period. Accordingly, we define the Coexisting- WBAN Parameter Period and Busy-channel Parameter Period as the duration of Coexisting-WBAN Parameter and the duration of Busy-channel Parameter, respectively.

State Period reflects the stability of coexisting WBANs. The State Period of the *i*-th state is signed as $T_{i}$. To illustrate the average and fluctuation of channel hopping frequency, we present Definitions 3 and 4.

**Definition** **3.** *In coexisting WBANs system, the expectation of Risk Parameter is called Coexisting Risk, which represents the average channel hopping frequency in the coexisting WBANs system. Coexisting Risk is signed as R*

(3) $$R=E(p)=\sum_{i=0}^{N}r_{i}p_{i}$$

**Definition** **4.** *In coexisting WBANs system, the variance of Risk Parameter is called Risk Variance, which represents the amplitude of channel hopping frequency. Risk Variance is signed as D*

(4) $$D=D\left(p\right)=\sum_{j=0}^{N}\left({\left(r_{j}-E\left(p\right)\right)}^{2}p_{j}\right)=\sum_{j=0}^{N}\left({\left(r_{j}-\sum_{i=0}^{N}r_{i}p_{i}\right)}^{2}p_{j}\right)$$

In coexisting WBANs system, there is a significant case that all the channels are occupied, leading to certainty channel conflict. We present Definition 5.

**Definition** **5.** As shown in Figure 4, the last state in state transition diagram is called Saturation State, the duration of Saturation State is called Saturation Period, and the probability of Saturation State is called Saturation Rate. Accordingly, we define Coexistence Saturation State as the state that the system can hold no more interfering WBANs, the duration of Coexistence Saturation State is called Coexistence Saturation Period, and the probability of coexistence saturation state is called Coexistence Saturation Rate. Similarly, we define Channel Saturation State as the state that all the channels are occupied, the duration of Channel Saturation State is called Channel Saturation Period, and the probability of Channel Saturation State is called Channel Saturation Rate.

The reason why we give Saturation-series definitions individually is to prevent the confusions when sensor nodes in a WBAN are allowed to be allocated to different channels. There is a different situation that the coexisting WBANs system can hold no more WBANs, but the channels are still unsaturated and more sensor nodes are allowed to be allocated to these channels. As described in Section 1 and Section 3.1.1, we treat WBAN as a minimum unit, (i.e., this paper investigates the coexistence capability at “WBAN level”). Unless otherwise specified, Saturation State, Coexistence Saturation State and Channel Saturation State are supposed to be equivalent in this level. Therefore, both Coexistence Saturation Rate and Channel Saturation Rate are equal to the probability of *N*-th state.

Coexistence Saturation Period and Coexistence Saturation Rate are significant metrics of coexisting WBANs. While there is another significant metric of interfering WBAN, referred to Definition 6.

**Definition** **6.** In coexisting WBANs system, the duration that the interfering WBAN occupies the channel is called Interfering Period.

Similarly, to investigate the individual channel usage situation, we present Channel Busy State, Channel Busy Period and Channel Busy Rate, referred to Definition 7.

**Definition** **7.** In coexisting WBANs system, the state that the channel can not hold another WBAN is called Channel Busy State and the duration of Channel Busy State is called Channel Busy Period. Furthermore, the probability Channel Busy State is called Channel Busy Rate.

Furthermore, when investigating the usage of channels, we provide Definition 8 and Theorem 1.

**Definition** **8.** In coexisting WBANs system, the ratio between occupied channels and specified channels is called Channel Utilization.

**Theorem** **1.** For coexisting WBANs system, Channel Utilization is equal to the average of Channel Busy Rate.

**Proof.**  Let $p_{c}\left(i\right)$ be the Channel Busy Rate of *i*-th channel. Due to the assumption that all channels are identical as described in Section 3.1.1, we obtain $p_{c}\left(1\right)=p_{c}\left(2\right)=\ldots =p_{c}\left(n\right)$. Let $p_{b}\left(k\right)$ be the probability that there are *k* busy channels. Because the channel allocation of interfering WBANs is independent identically distributed, the probability that the *i*-th channel is busy when there are *k* busy channels equals k/n. So the Channel Busy Rate of the *i*-th channel is

(5) $$p_{c}\left(i\right)=\sum_{k=0}^{n}\frac{k}{n}p_{b}\left(k\right)=\frac{1}{n}\sum_{k=0}^{n}kp_{b}\left(k\right)$$

The average number of busy channels is

(6) $$\bar{B}=\sum_{k=0}^{n}kp_{b}\left(k\right)$$

So the Channel Utilization is

(7) $$U=\frac{1}{n}\bar{B}=\frac{1}{n}\sum_{k=0}^{n}kp_{b}\left(k\right)$$

Therefore, $U=p_{c}\left(i\right)$, i.e., Channel Utilization is equal to Channel Busy Rate and Theorem 1 is proven. ☐

With definitions above we provided, the basic coexistence capability analysis construction is established for enhanced channel hopping mechanism. We can illustrate coexistence capability estimations for several classical coexisting WBANs.

## 5. Coexistence Capability Estimation

This section illustrates the coexistence capability analysis for enhanced channel hopping mechanism under both *1*-tolerated channel conflict model and *m*-tolerated channel conflict model.

### 5.1. Under 1-Tolerated Channel Conflict Model

In this part, we firstly discuss the basic property and theorems of 1-tolerated channel conflict model. Then we analyze the coexistence capability of the enhanced channel hopping mechanism taking into account queuing behaviors in Service-dependent coexisting WBANs and non-queuing behaviors in Service-independent coexisting WBANs, respectively. For the first one, we provide a Service-dependent analysis model. For the second one, we provide a Service-independent analysis model.

#### 5.1.1. Basic Property and Theorems

A basic property should be taken into account before we investigate the coexistence capability of the enhanced channel hopping mechanism under 1-tolerated channel conflict model, referred to Property 1.

**Property** **1.** In coexisting WBANs system under 1-tolerated channel conflict model, there is one-to-one correspondence between interfering WBANs and channels.

**Theorem** **2.** In coexisting WBANs system under 1-tolerated channel conflict model, Interfering Period is equal to Channel Busy Period.

**Proof.**  Under 1-tolerated channel conflict model, each interfering WBAN joins in the coexisting WBANs. If permitted, it will occupy a channel, and the channel turns to busy until the interfering WBAN leaves. So the Interfering Period is equal to Channel Busy Period, which proves Theorem 2. ☐

According to the queuing model assumption illustrated in Section 3.1.3 and general analysis model presented in Section 4.2, the Coexisting-WBAN Parameter $x_{k}$ is supposed to be *k*. Because *m* = 1 is the coexistence capacity of each channel, the maximum state index *N* is equal to *n*. Therefore, $S=\left\{\begin{array}{cccccccc} 0, & 1, & 2, & 3, & 4, & \ldots , & n-1, & n \end{array}\right\}$ and $X=\left\{\begin{array}{cccccccc} 0, & 1, & 2, & 3, & 4, & \ldots , & n-1, & n \end{array}\right\}$. Furthermore, the correlation between Coexisting-WBAN Parameter and Busy-channel Parameter is referred to Theorem 3.

**Theorem** **3.** In coexisting WBANs under 1-tolerated channel conflict model, Coexisting-WBAN Parameter *X* and Busy-channel Parameter *Y* have the same state space and probability distribution.

**Proof.**  According to Property 1, for any $x_{k}=k$, $y_{k}=k$ and $r_{k}=k/n$. So *X* and *Y* have the same state space. According to Figure 4, the state-parameter pairs *(X, Y)* have the same probability distribution with S. Therefore, *X* and *Y* also have the same probability distribution, as required. ☐

The advantage of *X* and *Y* assumption here is that we can employ M/M/c/n/∞ queuing models as our analysis basis, in which c represents the number of servers, n represents the system capacity, ∞ indicates that the customers are infinite. In Service-dependent analysis model, *c* equals 1. While in the Service-independent analysis model, *c* equals the channel coexistence capacity *m*.

#### 5.1.2. Service-Dependent Analysis Model

As mentioned above, when taking into account queuing behavior, the Service-dependent coexisting WBANs (as shown in Figure 3a) with arriving rate $\lambda_{k}$ and service rate $\mu_{k}$ (as shown in Equations (8) and (9)), is exactly a M/M/1/N/*∞*-based queuing system model.

(8) $$\lambda_{k}=\left\{\begin{array}{c} \lambda \begin{array}{ccc} & & k=0,1,\ldots ,n-1 \end{array}\\ 0\begin{array}{ccc} & & k\geq n \end{array} \end{array}\right.$$

(9) $$\mu_{k}=\left\{\begin{array}{c} \mu \begin{array}{ccc} & & k=1,2,\ldots ,n \end{array}\\ 0\begin{array}{ccc} & & k>n \end{array} \end{array}\right.$$

**Figure 5:**
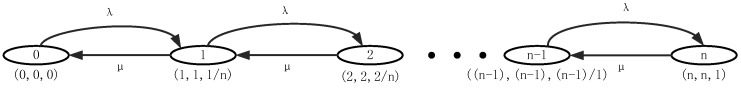
State transition diagram for enhanced channel hopping mechanism in Service-dependent coexisting WBANs.

Therefore, the state transition diagram can be illustrated as Figure 5.

The equilibrium equations are

(10) $$\left\{\begin{array}{c} \lambda p_{0}=\mu p_{1}\\ -\left(\lambda +\mu \right)p_{k}+\lambda p_{k-1}+\mu p_{k+1}=0\begin{array}{ccc} & & \left(k=1,2,\ldots ,n-1\right) \end{array}\\ \lambda p_{n-1}=\mu p_{n} \end{array}\right.$$

We set $\rho =\frac{\lambda }{\mu }$, so $p_{k}=\rho^{k}p_{0}$. According to the probabilistic characteristics, $\sum_{k=0}^{n}p_{k}\;=\;p_{0}\;+\;\rho p_{0}+\ldots +\rho^{k}p_{0}\ldots +\;\rho^{n}p_{0}\;=\;p_{0}\frac{1-\rho^{n+1}}{1-\rho }=1$, we obtain $p_{0}=\frac{1-\rho }{1-\rho^{n+1}}$. Then the steady-state distribution can be written as

(11) $$\left\{\begin{array}{c} p_{0}=\frac{1-\rho }{1-\rho^{n+1}}\\ p_{k}=\frac{\left(1-\rho \right)\rho^{k}}{1-\rho^{n+1}}\begin{array}{ccc} & & \left(k=1,\ldots ,n\right) \end{array} \end{array}\right.$$

According to Theorem 3, the probability that there are *k* busy channels is equal to the state probability $p_{k}$, signed as $p_{b}\left(k\right)$. Therefore we can obtain the Saturation Rate, Saturation Period, Coexisting Risk and Risk Variance, referring to Lemmas 1–4.

**Lemma** **1.** The Saturation Rate in Service-dependent coexisting WBANs is $\frac{\left(1-\rho \right)\rho^{n}}{1-\rho^{n+1}}$, so do Coexistence Saturation Rate and Channel Saturation Rate.

**Proof.**  According to Definition 5 and Equation (11), the saturation rate

(12) $$p_{ds}=p_{n}=\frac{\left(1-\rho \right)\rho^{n}}{1-\rho^{n+1}}$$

According to the general analysis model and Definition 5 presented in Section 4.2, Saturation State, Coexistence Saturation State and Channel Saturation State are supposed to be equivalent in this paper. The Coexistence Saturation Rate and Channel Saturation Rate are also equal to $\frac{\left(1-\rho \right)\rho^{n}}{1-\rho^{n+1}}$, as required. ☐

**Lemma** **2.** The average of Saturation Period in Service-dependent coexisting WBANs is $\frac{1}{\mu }$, so do Coexistence Saturation Period and Channel Saturation Period.

**Proof.**  According to the memoryless property of negative exponential distribution, the duration before the coming event occurrence also follows negative exponential distribution with the same parameter. In Service-dependent coexisting WBANs, the service rate of state *N* is *μ*. So the average of Saturation Period is $\frac{1}{\mu }$. Similar to Lemma 1, Saturation State, Coexistence Saturation State and Channel Saturation State are supposed to be equivalent. The Coexistence Saturation Period and Channel Saturation Period are also equal to $\frac{1}{\mu }$, as required. ☐

**Lemma** **3.** The Coexisting Risk in Service-dependent coexisting WBANs is $\sum_{i=0}^{n}\frac{i}{n}\rho^{i}\frac{1-\rho }{1-\rho^{n+1}}$.

**Proof.**  According to Definition 3, Coexisting Risk is

(13) $$R_{d}=E\left(p\right)=\sum_{i=0}^{n}r_{i}p_{i}=\sum_{i=0}^{n}\frac{i}{n}\rho^{i}\frac{1-\rho }{1-\rho^{n+1}}$$

as required. ☐

**Lemma** **4.** *The Risk Variance in Service-dependent coexisting WBANs is*

$$\sum_{j=0}^{n}\left({\left(\frac{j}{n}-\sum_{i=0}^{n}\frac{i}{n}\rho^{i}\frac{1-\rho }{1-\rho^{n+1}}\right)}^{2}\rho^{j}\frac{1-\rho }{1-\rho^{n+1}}\right)$$

**Proof.**  According to Definition 4, Risk Variance is

(14) $$\begin{array}{c} D_{d}=D\left(p\right)\\ \;\;\;\;\;=\sum_{j=0}^{n}\left({\left(r_{j}-E\left(p\right)\right)}^{2}p_{j}\right)\\ \;\;\;\;\;=\sum_{j=0}^{n}\left({\left(\frac{j}{n}-\sum_{i=0}^{n}\frac{i}{n}\rho^{i}\frac{1-\rho }{1-\rho^{n+1}}\right)}^{2}\rho^{j}\frac{1-\rho }{1-\rho^{n+1}}\right) \end{array}$$

as required. ☐

**Lemma** **5.** The Channel Utilization and the average of Channel Busy Rate in Service-dependent coexisting WBANs are $\frac{\rho }{n\left(1-\rho \right)}-\frac{\left(n+1\right)\rho^{k+1}}{n\left(1-\rho^{k+1}\right)}$.

**Proof.**  The expected value of queue length is

(15) $$L_{d}=\sum_{k=0}^{n}kp_{k}=\sum_{k=0}^{n}k\frac{\left(1-\rho \right)\rho^{k}}{1-\rho^{n+1}}$$

Because there is a one-to-one correspondence property between a WBAN and the channel in which it joins under 1-tolerated channel conflict model. The queue length represents the number of busy channels. According to Definition 8 and Theorem 1, the Channel Utilization and Channel Busy Rate are

(16) $$U_{d}=P_{B}=\frac{L}{n}=\frac{1}{n}\sum_{k=0}^{n}k\frac{\left(1-\rho \right)\rho^{k}}{1-\rho^{n+1}}$$

as required. ☐

**Lemma** **6.** *The average of Interfering Period and the average of Channel Busy Period are equal to*

$$\frac{1}{\lambda \left(1-\frac{\left(1-\rho \right)\rho^{n}}{1-\rho^{n+1}}\right)}\sum_{k=0}^{n}k\frac{\left(1-\rho \right)\rho^{k}}{1-\rho^{n+1}}$$

**Proof.**  According to Little Law in Queuing Theory, the residence time of the consumer is $W_{s}=\frac{L_{s}}{\lambda_{e}}$, where $\lambda_{e}=\lambda \left(1-p_{n}\right)$ is the effective arriving rate. So the average of Interfering Period $T_{C}$ is

(17) $$T_{C}=W_{S}=\frac{L_{S}}{\lambda_{e}}=\frac{1}{\lambda \left(1-\frac{\left(1-\rho \right)\rho^{n}}{1-\rho^{n+1}}\right)}\sum_{k=0}^{n}k\frac{\left(1-\rho \right)\rho^{k}}{1-\rho^{n+1}}$$

As Channel Busy Period equals Interfering Period under 1-tolerated channel conflict model according to Theorem 2, Lemma 6 is proven. ☐

#### 5.1.3. Service-Independent Analysis Model

Different from Service-dependent coexisting WBANs, the interfering WBANs leave the coexisting WBANs without any queuing constraint in Service-independent coexisting WBANs as shown in Figure 3b. Then the arriving rate and service rate are written as Equations (18) and (19), respectively, indicating that the coexisting WBANs system is exactly an M/M/n/n/∞ queuing system.

(18) $$\lambda_{k}=\left\{\begin{array}{c} \lambda \begin{array}{ccc} & & k=0,1,\ldots ,n-1 \end{array}\\ 0\begin{array}{ccc} & & k\geq n \end{array} \end{array}\right.$$

(19) $$\mu_{k}=\left\{\begin{array}{c} k\mu \;\;\;\;\;\;\;\;k=1,2,\ldots ,n\\ 0\;\;\;\;\;\;\;\;\;\;k>n \end{array}\right.$$

**Figure 6:**
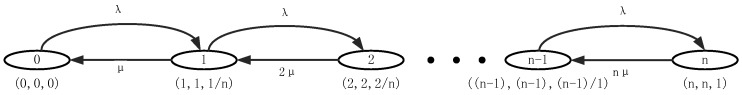
Service-independent state transition diagram for enhanced channel hopping mechanism in coexisting WBANs.

Then the state transition diagram can be illustrated as Figure 6.

The equilibrium equations are

(20) $$\left\{\begin{array}{c} \lambda p_{0}=\mu p_{1}\\ -\left(\lambda +k\mu \right)p_{k}+\lambda p_{k-1}+\left(k+1\right)\mu p_{k+1}=0\begin{array}{ccc} & & \left(k=1,2,\ldots ,n-1\right) \end{array}\\ \lambda p_{n-1}=n\mu p_{n} \end{array}\right.$$

So $p_{1}=\frac{\lambda }{\mu }p_{0},p_{2}=\frac{\lambda }{2\mu }p_{1},\ldots ,p_{n}=\frac{\lambda }{n\mu }p_{n-1}$, then $p_{k}=\frac{1}{k!}{\left(\frac{\lambda }{\mu }\right)}^{k}p_{0},k=0,1,2,\ldots ,n$. As $\rho =\frac{\lambda }{\mu }$, $p_{k}=\frac{\rho^{k}}{k!}p_{0},k=0,1,2,\ldots ,n$. According to the probabilistic characteristics, $\sum_{k=0}^{n}p_{k}=1$, we obtain $p_{0}=\frac{1}{\sum_{r=0}^{n}\frac{\rho^{r}}{r!}}$. Then the steady-strate distribution can be written as

(21) $$p_{k}=\frac{\rho^{k}/k!}{\sum_{r=0}^{n}\frac{\rho^{r}}{r!}},k=0,1,2,\ldots ,n$$

**Lemma** **7.** The Saturation Rate, Coexistence Saturation Rate and Channel Saturation Rate in Service-independent coexisting WBANs are all equal to $\frac{\rho^{n}/n!}{\sum_{r=0}^{n}\frac{\rho^{r}}{r!}}$.

**Proof.**  According to Definition 5 and Equation (21), the Coexistence Saturation Rate is

(22) $$p_{ins}=p_{n}=\frac{\rho^{n}/n!}{\sum_{r=0}^{n}\frac{\rho^{r}}{r!}}$$

Similar to Lemma 1, because Saturation State, Coexistence Saturation State and Channel Saturation State are assumed to be equivalent, the Coexistence Saturation Rate and Channel Saturation Rate are also equal to $\frac{\rho^{n}/n!}{\sum_{k=0}^{n}\frac{\rho^{n}}{n!}}$, as required. ☐

**Lemma** **8.** The average of Saturation Period, the average of Coexistence Saturation Period and the average of Channel Saturation Period in Service-independent coexisting WBANs are all equal to $\frac{1}{n\mu }$.

**Proof.**  Similar to the proof in Lemma 2, according to the memoryless property of negative exponential distribution, the duration between the arriving and leaving events follows negative exponential distribution. In Service-independent coexisting WBANs, the leaving rate of state *N* is nμ. So the average of Saturation Period is $\frac{1}{n\mu }$. Similar to Lemmas 1 and 7, Saturation State, Coexistence Saturation State and Channel Saturation State are assumed to be equivalent. The Coexistence Saturation Period and Channel Saturation Period are also equal to $\frac{1}{n\mu }$, as required. ☐

**Lemma** **9.** The Coexisting Risk in Service-independent coexisting WBANs is $\sum_{i=0}^{n}\frac{i}{n}\frac{\rho^{i}/i!}{\sum_{r=0}^{n}\frac{\rho^{r}}{r!}}$.

**Proof.**  According to Definition 3, Coexisting Risk is

(23) $$R_{in}=E\left(p\right)=\sum_{i=0}^{n}r_{i}p_{i}=\sum_{i=0}^{n}\frac{i}{n}\frac{\rho^{i}/i!}{\sum_{r=0}^{n}\frac{\rho^{r}}{r!}}$$

as required. ☐

**Lemma** **10.** *The Risk Variance in Service-independent coexisting WBANs is*

$$\sum_{j=0}^{n}\left({\left(\frac{j}{n}-\sum_{i=0}^{n}\frac{i}{n}\frac{\rho^{i}/i!}{\sum_{r=0}^{n}\frac{a^{r}}{r!}}\right)}^{2}\frac{\rho^{j}/j!}{\sum_{r=0}^{n}\frac{a^{r}}{r!}}\right)$$

**Proof.**  According to Definition 4, Risk Variance is

(24) $$D_{in}=D\left(p\right)=\sum_{j=0}^{n}\left({\left(r_{j}-E\left(p\right)\right)}^{2}p_{j}\right)=\sum_{j=0}^{n}\left({\left(\frac{j}{n}-\sum_{i=0}^{n}\frac{i}{n}\frac{\rho^{i}/i!}{\sum_{r=0}^{n}\frac{a^{r}}{r!}}\right)}^{2}\frac{\rho^{j}/j!}{\sum_{r=0}^{n}\frac{a^{r}}{r!}}\right)$$

as required. ☐

**Lemma** **11.** The Channel Utilization and Channel Busy Rate in Service-independent coexisting WBANs are $\frac{1}{n}\sum_{i=0}^{n}\frac{\rho^{k}/\left(k-1\right)!}{\sum_{r=0}^{n}\frac{\rho^{r}}{r!}}$

**Proof.**  The expected value of queue length is

(25) $$L_{in}=\sum_{k=0}^{n}kp_{k}=\sum_{i=0}^{n}\frac{\rho^{k}/\left(k-1\right)!}{\sum_{r=0}^{n}\frac{\rho^{r}}{r!}}$$

According to Definition 8 and Theorem 1, the Channel Utilization and the average of Channel Busy Rate are

(26) $$U_{in}=P_{B}=\frac{L}{n}=\frac{1}{n}\sum_{i=0}^{n}\frac{\rho^{k}/\left(k-1\right)!}{\sum_{r=0}^{n}\frac{\rho^{r}}{r!}}$$

as required. ☐

**Lemma** **12.** The average of Interfering Period and the average of Channel Busy Period in Service-independent coexisting WBANs are $\frac{1}{\mu }$.

**Proof.**  As there is no queuing waiting in Service-independent coexisting WBANs, we can simply consider service time $\frac{1}{\mu }$ as Interfering Period. According to Theorem 2, the average of Channel Busy Period is equal to the average Interfering Period, as required. ☐

As analyzed above, a set of important metrics are provided for coexistence capability estimation under 1-tolerated channel conflict model.

### 5.2. Under m-Tolerated Channel Conflict Model

Different from 1-tolerated channel conflict model, under which Coexisting-WBAN Parameter and Busy-channel Parameter have the same state space and probability distribution, the Busy-channel Parameter *Y* under *m*-tolerated channel conflict model not only depends on the number of interfering WBANs, but also depends on the distribution of channel allocation. It is hard to provide a theoretical solution for Busy-channel Parameter probability distribution estimation under *m*-tolerated channel conflict models. This paper presents two approximate methods to estimate the Busy-channel Parameter. The first one is based on a basic assumption that channel allocations of interfering WBANs are randomly and follow uniform distribution. The second one is based on the additive property of Possion stream that a Possion stream can be divided into multiple Possion substreams. As analyzed in Section 5.1, we can easily obtain the Coexisting-WBAN Parameter distribution in 1-tolerated channel conflict model, which can also be applied to *m*-tolerated channel conflict model. Therefore, this part does not represent the estimation procedure of Coexisting-WBAN Parameter in the first approximate method. While in the second approximate method, Coexisting-WBAN Parameter is not respected.

#### 5.2.1. Approximate Method Based on Uniform Distribution Assumption

This part still supposes that the Coexisting-WBAN Parameter $x_{k}=k$. Because the coexistence capacity of each channel *m* > 1, the maximum coexisting state N=m·n>n. Therefore, $X\;=\;\left\{\begin{array}{cccccccc} 0, & 1, & 2, & 3, & 4, & \ldots , & m\cdot n-1, & m\cdot n \end{array}\right\}$, but $y_{k}$ is not unique but an interval. This paper signs $y_{k}\left(i\right)$ as there are *i* busy channels when *k* WBANs are in the coexisting system. The Busy-channel Parameter distributions of each $y_{k}$ are written as $\bar{y_{k}}$. Accordingly, the Risk Parameter distribution is written as $\bar{r_{k}}$, which equals $\bar{y_{k}}/n$.

The value interval of $y_{k}$ is

(27) $$\left\{\begin{array}{c} y_{k}=0,\;\;\;\;\;\;\;\;\;\;\;\;\;\;\;\;\;\;\;\;\;\;\;\;\;\;\;\;k<m\\ \left\lfloor k/n\right\rfloor \leq y_{k}\leq \left\lfloor k/m\right\rfloor ,\;\;\;\;\;m\leq k\leq m\cdot n\\ y_{m\cdot n}=n,\;\;\;\;\;\;\;\;\;\;\;\;\;\;\;\;\;\;\;\;\;\;\;\;k=m\cdot n \end{array}\right.$$

As $r_{k}=y_{k}/n$, the value interval of $r_{k}$ is

(28) $$\left\{\begin{array}{c} r_{k}=0,\;\;\;\;\;\;\;\;\;\;\;\;\;\;\;\;\;\;\;\;\;\;\;\;\;\;k<m\\ \frac{1}{\left\lfloor k/m\right\rfloor }\leq r_{k}\leq \frac{1}{\left\lfloor k/n\right\rfloor },\;\;\;\;\;\;m\leq k\leq m\cdot n\\ r_{m\cdot n}=1,\;\;\;\;\;\;\;\;\;\;\;\;\;\;\;\;\;\;\;\;\;\;k=m\cdot n \end{array}\right.$$

**Figure 7:**
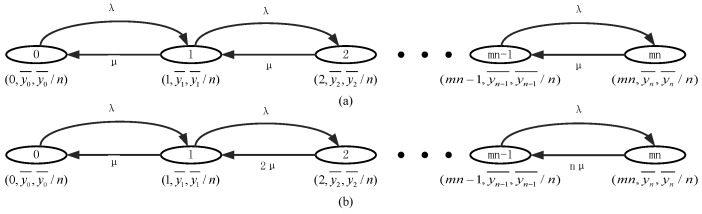
State transition diagrams of coexisting WBANs under m-tolerated channel conflict model. (**a**) Service-dependent analysis model; (**b**) Service-independent analysis model.

The state transition diagram can be illustrated as Figure 7.

**Figure 8:**
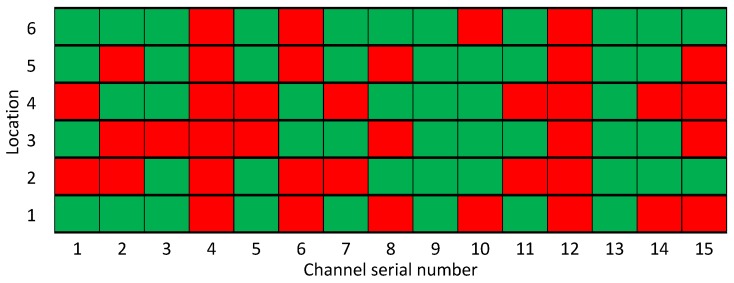
Channel occupied bit map illustration for *m*-tolerate coexistence WBANs.

Assuming that the channel allocation is following uniform distribution, we employ a channel occupied bit map as shown in Figure 8. The horizontal axis is the specified channels and the vertical axis is the coexistence capacity of each channel for interfering WBANs. The red bit denotes the location is occupied and the green bit denotes the location is free. 4-th and 12-th channel indicate that the channels are busy/occupied.

We sign $p_{b}\left(n_{1},k,m,n\right)$ as the probability that there are $n_{1}$ busy channels with *k* WBANs in a *m × n* coexisting system. We employ the recursive function $p_{b}\left(n_{1},k,m,n\right)$ to obtain the number of legal cases.

(29) $$f\left(n_{1},k,n,m\right)=\left\{\begin{array}{ll} 0, & k<m\cdot n_{1}\\ A_{m\cdot n}^{k}, & k<m\;and\;n_{1}=0\\ C_{n}^{n_{1}}A_{k}^{m\cdot n_{1}}\left(A_{m\left(n-n_{1}\right)}^{k-m\cdot n_{1}}-\sum_{i=0}^{n-n_{1}}f\left(i,k-m\cdot n_{1},n-m\cdot n_{1},m\right)\right), & k\geq m\cdot n_{1} \end{array}\right.$$

Then we can get $\bar{y_{k}}$ through

(30) $$p_{b}\left(n_{1},k,n,m\right)=\frac{f\left(n_{1},k,n,m\right)}{A_{m\cdot n}^{k}}$$

Further, we can obtain the global Busy-channel Parameter distribution, written as

(31) $$p_{bc}=\sum_{k=0}^{m\cdot n}\bar{y_{k}}.*p_{k}$$

Accordingly, the Risk Parameter is written as RP={1,1/2,1/3,…,1/n}. Then the Coexisting Risk is

(32) $$R=\sum RP.*p_{bc}$$

The Risk Variance is

(33) $$D=\sum \left(RP-R\right).*\left(RP-R\right).*p_{bc}$$

Similar to Section 5.1, we investigate coexistence capability of the enhanced channel hopping mechanism under *m*-tolerated channel conflict model by taking into account queuing and non-queuing behaviors, respectively.

According to Theorem 1, the Channel Busy Rate and Channel Utilization in both Service-dependent coexisting WBANs and Service-independent coexisting WBANs can be written as

(34) $$U=\frac{1}{n}R=\frac{1}{n}\sum RP.*p_{bc}$$

From the global Busy-channel Parameter distribution $p_{bc}$, it is easy to obtain Coexistence Saturation Rate and Channel Saturation Rate. Here we give another expression, because the general analysis model in Section 4.2 has already been adopted in this approximate method.

According to the queuing model assumption illustrated in Section 3.1.3, the Saturation-series definitions and general analysis model presented in Section 4.2, the Coexistence Saturation Rate in both Service-dependent and Service-independent coexisting WBANs under *m*-tolerated channel conflict model are equal to their Saturate Rate, as well as that under 1-tolerated channel conflict model. According to Lemma 1, the Coexistence Saturation Rate and Channel Saturation Rate in Service-dependent coexisting WBANs and Service-independent coexisting WBANs are written as Equations (35) and (36), respectively.

(35) $$p_{mds}=p\left(N\right)=p\left(m\cdot n\right)=\frac{\left(1-\rho \right)\rho^{m\cdot n}}{1-\rho^{m\cdot n+1}}$$

(36) $$p_{mins}=p\left(N\right)=p\left(m\cdot n\right)=\frac{\frac{\rho^{m\cdot n}}{\left(m\cdot n\right)!}}{\sum_{k=0}^{m\cdot n}\frac{\rho^{m\cdot n}}{\left(m\cdot n\right)!}}$$

According to Lemmas 2 and 8, the average of Saturation Period, the average of Coexistence Saturation Period and the average of Channel Saturation Period in Service-dependent coexisting WBANs and Service-independent coexisting WBANs all are equal to $\frac{1}{\mu }$ and $\frac{1}{m\cdot n\cdot \mu }$, respectively.

According to Lemma 6, the average Interfering Period in Service-dependent coexisting WBANs and Service-independent coexisting WBANs are equal to Equation (37) and $\frac{1}{\mu }$, respectively.

(37) $$T_{C}=\frac{1}{m\cdot n\left(1-\frac{\left(1-\rho \right)\rho^{m\cdot n}}{1-\rho^{m\cdot n+1}}\right)}\sum_{k=0}^{m\cdot n}k\frac{\left(1-\rho \right)\rho^{k}}{1-\rho^{m\cdot n+1}}$$

Consequently, we can obtain the key parameters and metrics of coexistence capability from this approximate method.

#### 5.2.2. Approximate Method Based on Additive Property of Possion-Stream

This part provides another coexistence capability approximate method. We notice that there is a special additive property of Possion stream, refer to Property 2. Different from the estimation methods we provided in Section 5.1 and Section 5.2.1, the Coexisting-WBAN Parameter in general analysis model is not respected here, and we only focus on the estimation of Busy-channel Parameter. From Property 2, we obtain Lemma 13.

**Property** **2.** A Possion stream with λ=$\lambda_{1}+\lambda_{2}+\lambda_{3}+\ldots +\lambda_{n}$ can be divided into *n* independent Possion substreams with parameters $\lambda_{1},\lambda_{2},\lambda_{3},\ldots ,\lambda_{n}$, respectively.

**Lemma** **13.** *A variable* Ω *which follows negative exponential distribution with parameter μ can be divided into *n* variables $\left\{\omega_{1},\omega_{2},\ldots \omega_{n}\right\}$, which are independent variables following negative exponential distributions with parameters $\left\{\mu_{1},\mu_{2},\ldots ,\mu_{n}\right\}$ respectively, where $\mu =\mu_{1}+\mu_{2}+\ldots +\mu_{n}$.*

**Proof.**  Assuming that the occurrence times of event A within unit time are following Possion distribution with parameter *μ*, the duration of each neighboring events is following negative exponential distribution with parameter *μ*. According to Lemma 2, the Possion stream can be divided into *n* substream with parameters $\left\{\mu_{1},\mu_{2},\ldots ,\mu_{n}\right\}$, and $\mu =\mu_{1}+\mu_{2}+\ldots +\mu_{n}$. The duration of each neighboring events in each substream is following negative exponential distribution with parameter $\mu_{k}\in \left\{\mu_{1},\mu_{2},\ldots ,\mu_{n}\right\}$. Lemma 13 is proven. ☐

**Figure 9:**
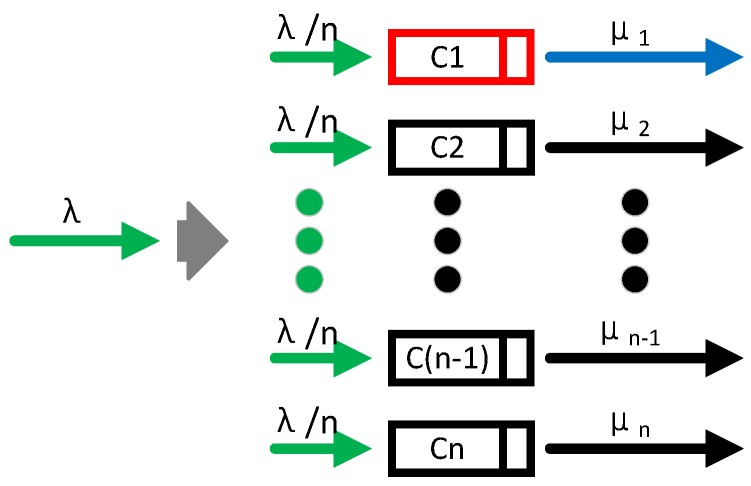
Coexistence capability analysis with n independent channels based on additive property of Possion-stream.

According to Property 2, the Possion stream can be divided into *n* Possion substreams with $\lambda_{1}=\lambda_{2}=\ldots =\lambda_{n}=\frac{\lambda }{n}$, as shown in Figure 9. Ck is considered as an individual queuing system as shown in Figure 3a with queuing behavior and as shown in Figure 3b with non-queuing behavior, respectively. According to Lemma 13, the leaving rate in Service-dependent coexisting WBANs is μ/n, but in Service-independent coexisting WBANs is *μ*.

**Figure 10:**
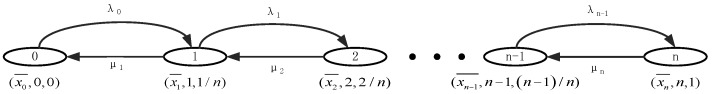
State transition diagram of coexisting WBANs under m-tolerated channel conflict model.

The state transition diagram can be illustrated as Figure 10. $\bar{x_{k}}$ is considered as the distribution of Coexisting-WBAN Parameter, which can be easily estimated based on Bayesian Theory if necessary. Here, we do not respect the Coexisting-WBAN Parameter, as well as $\lambda_{k}$ and $\mu_{k}$.

As each channel can be considered as an individual coexisting system, the probability that the channel is busy equals the Coexistence Saturation Rate of Ck. According to Lemmas 1 and 7 and Theorem 1, the Channel Busy Rate and Channel Utilization in Service-dependent coexisting WBANs and Service-independent coexisting WBANs are Equations (38) and (39), respectively.

(38) $$p_{mds}=\frac{\left(1-\rho \right)\rho^{m}}{1-\rho^{m+1}}$$

(39) $$p_{mins}=\frac{\frac{\rho^{m}}{m!}}{\sum_{r=0}^{m}\frac{\rho^{r}}{r!}}$$

Thus the probability that there are $n_{1}$ busy channel in Service-dependent coexisting WBANs and Service-independent coexisting WBANs are approximate to Equations (40) and (41), respectively.

(40) $$\begin{array}{c} p_{md}\left(n_{1}\right)=C_{n}^{n_{1}}p_{mds}^{n_{1}}{\left(1-p_{mds}\right)}^{n-n_{1}}\\ \;\;\;\;\;\;\;\;\;\;\;\;\;=\frac{n!}{n_{1}!\left(n-n_{1}\right)!}p_{cbr}^{n_{1}}{\left(1-p_{cbr}\right)}^{n-n_{1}}\\ \;\;\;\;\;\;\;\;\;\;\;\;\;=\frac{n!}{n_{1}!\left(n-n_{1}\right)!}\frac{{\left(1-\rho \right)}^{n_{1}}\rho^{m\cdot n_{1}}{\left(1-\rho^{m}\right)}^{n-n_{1}}}{{\left(1-\rho^{m+1}\right)}^{n}} \end{array}$$

(41) $$\begin{array}{c} p_{min}\left(n_{1}\right)=C_{n}^{n_{1}}p_{mins}^{n_{1}}{\left(1-p_{mins}\right)}^{n-n_{1}}\\ \;\;\;\;\;\;\;\;\;\;\;\;\;\;=\frac{n!}{n_{1}!\left(n-n_{1}\right)!}p_{mins}^{n_{1}}{\left(1-p_{mins}\right)}^{n-n_{1}}\\ \;\;\;\;\;\;\;\;\;\;\;\;\;\;=\frac{n!}{n_{1}!\left(n-n_{1}\right)!}\frac{{\left(\frac{\rho^{m}}{m!}\right)}^{n_{1}}{\left(\sum_{r=0}^{m}\frac{\rho^{r}}{r!}-\frac{\rho^{m}}{m!}\right)}^{n-n_{1}}}{{\left(\sum_{r=0}^{m}\frac{\rho^{r}}{r!}\right)}^{n}} \end{array}$$

The Coexistence Saturation Rate in Service- dependent coexisting WBANs and Service-independent coexisting WBANs are approximate to Equations (42) and (43), respectively.

(42) $$p_{md}\left(n\right)={\left(\frac{\left(1-\rho \right)\rho^{m}}{1-\rho^{m+1}}\right)}^{n}$$

(43) $$p_{min}\left(n\right)={\left(\frac{\frac{\rho^{m}}{m!}}{\sum_{r=0}^{m}\frac{\rho^{r}}{r!}}\right)}^{n}$$

According to Definition 3, the Coexisting Risk in Service-dependent coexisting WBANs and Service-independent coexisting WBANs are approximate to Equations (44) and (45), respectively.

(44) $$R_{d}=\sum_{i=0}^{n}r_{i}p_{md}\left(i\right)=\sum_{i=0}^{n}\frac{i}{n}\frac{n!}{i!\left(n-i\right)!}\frac{{\left(1-\rho \right)}^{i}\rho^{m\cdot i}{\left(1-\rho^{m}\right)}^{n-i}}{{\left(1-\rho^{m+1}\right)}^{n}}$$

(45) $$R_{in}=\sum_{i=0}^{n}r_{i}p_{min}\left(i\right)=\sum_{i=0}^{n}\frac{i}{n}\frac{n!}{i!\left(n-i\right)!}\frac{{\left(\frac{\rho^{m}}{m!}\right)}^{i}{\left(\sum_{r=0}^{m}\frac{\rho^{r}}{r!}-\frac{\rho^{m}}{m!}\right)}^{n-i}}{{\left(\sum_{r=0}^{m}\frac{\rho^{r}}{r!}\right)}^{n}}$$

According to Definition 4, the Risk Variance in Service-dependent coexisting WBANs and Service-independent coexisting WBANs are approximate to Equations (46) and (47), respectively.

(46) $$\begin{array}{c} D_{d}=\sum_{k=0}^{n}\left({\left(r_{k}-R_{d}\right)}^{2}p_{md}\left(k\right)\right)\\ \;\;\;\;\;=\sum_{k=0}^{n}\left({\left(\frac{k}{n}-\sum_{i=0}^{n}\frac{i}{n}\frac{n!}{i!\left(n-i\right)!}\frac{{\left(1-\rho \right)}^{i}\rho^{mi}{\left(1-\rho^{m}\right)}^{n-i}}{{\left(1-\rho^{m+1}\right)}^{n}}\right)}^{2}\frac{n!}{k!\left(n-k\right)!}\frac{{\left(1-\rho \right)}^{k}\rho^{m\cdot k}{\left(1-\rho^{m}\right)}^{n-k}}{{\left(1-\rho^{m+1}\right)}^{n}}\right) \end{array}$$

(47) $$\begin{array}{c} D_{in}=\sum_{k=0}^{n}\left({\left(r_{k}-R_{in}\right)}^{2}p_{min}\left(k\right)\right)\\ \;\;\;\;\;\;=\sum_{k=0}^{n}\left({\left(\frac{k}{n}-\sum_{i=0}^{n}\frac{i}{n}\frac{n!}{i!\left(n-i\right)!}\frac{{\left(\frac{\rho^{m}}{m!}\right)}^{i}{\left(\sum_{r=0}^{m}\frac{\rho^{r}}{r!}-\frac{\rho^{m}}{m!}\right)}^{n-i}}{{\left(\sum_{r=0}^{m}\frac{\rho^{r}}{r!}\right)}^{n}}\right)}^{2}\frac{n!}{k!\left(n-k\right)!}\frac{{\left(\frac{\rho^{m}}{m!}\right)}^{k}{\left(\sum_{r=0}^{m}\frac{\rho^{r}}{r!}-\frac{\rho^{m}}{m!}\right)}^{n-k}}{{\left(\sum_{r=0}^{m}\frac{\rho^{r}}{r!}\right)}^{n}}\right) \end{array}$$

According to Lemmas 6 and 13, the average of Channel Busy Period in Service-dependent coexisting WBANs is

$$\frac{n}{\lambda \left(1-\frac{\left(1-\rho \right)\rho^{m}}{1-\rho^{m+1}}\right)}\sum_{k=0}^{m}k\frac{\left(1-\rho \right)\rho^{k}}{1-\rho^{m+1}}$$

where $\rho =\frac{\lambda }{\mu }$. According to Lemmas 12 and 13, the average of Channel Busy Period in Service-independent coexisting WBANs is equal to $\frac{1}{\mu }$. According to Lemmas 2 and 8, the average of Saturation Period, the average of Coexistence Saturation Period and the average of Channel Saturation Period in Service-dependent coexisting WBANs and Service-independent coexisting WBANs all are equal to $\frac{1}{\mu }$ and $\frac{1}{m\cdot n\cdot \mu }$, respectively.

Consequently, parameters and metrics of coexistence capability in both Service-dependent and Service-independent coexisting WBANs could be obtained through our analysis. For *m*-tolerated channel conflict model, most significant parameters and metrics can be easily achieved by our approximate methods.

## 6. Simulation Results and Discussion

This section provides extensive simulation results to verify our mechanism and coexistence capability analysis models. Firstly, the coexistence capability parameters evaluations are presented, including Coexisting-WBAN Parameter and Busy-channel Parameter. Then coexistence capability metrics are evaluated, such as Coexisting Risk, Risk Variance, Channel Saturation Rate and Interfering Period. At last, channel conflict times in each time unit are employed to reflect the coexistence performance of channel hopping mechanism. Both Service-dependent and Service-independent coexistence models are taken into account in our simulations.

The parameters in our simulations are illustrated in Table 3. According to IEEE 802.15.6, which specifies different number of channels (10, 12, 14, 16, 39, 60, 79) for coexisting WBANs, we employ the worst case for coexistence capability studies, i.e., n=10. Without loss of generality, we employ one second as the basic time unit for channel conflict counting. To investigate the coexistence capability of the enhanced channel hopping mechanism, we employ {1, 2, 3, 4, 5, 6} as our channel coexistence capacity. As described in Section 3.1.1, 1-tolerated channel conflict model represents the plain channel hopping mechanism which does not integrate other channel-shared coexistence technologies. The advantages of enhanced channel hopping mechanism will be demonstrated in this section.

### 6.1. Coexistence Capability Parameters Discussion

In this part, we investigate the probability distribution of Coexisting-WBAN/Busy-channel Parameter under 1-tolerated channel conflict model and investigate the probability distribution of Busy-channel Parameter under m-tolerated channel conflict model. In our simulation, we employ the ratio between the total period of each state and the whole simulation period as Period Rate, and employ the ratio between the times of each state appears and the total state transition times as Frequency Rate.

#### 6.1.1. 1-Tolerated Channel Conflict Model

In 1-tolerated channel conflict model, the Coexisting-WBAN Parameter and Busy-channel Parameter are equivalent, as illustrated in Theorem 3.

**Figure 11:**
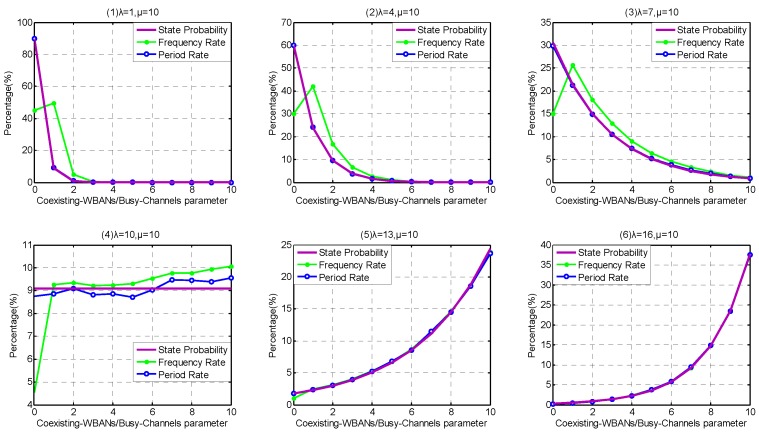
Coexisting-WBAN/Busy-channel Parameter probability distribution under 1-tolerated channel conflict model in Service-dependent coexisting WBANs.

Firstly, because the coexistence capability in Service-dependent coexisting WBANs are weak, we investigate the influence of different arriving rates *λ* within the interval [1, 16]. We set the step to be 3, so λ= {1, 4, 7, 10, 13, 16}. As shown in Figure 11, the Period Rate is closer to state probability distribution than Frequency Rate, especially the front several states. This is because the service rate of state 0 is zero, resulting in the state stagnating until the next WBAN arrives. Along with the state increase, the Period Rate and Frequency Rate approach each other, which means that the influence of state 0 is weakened. From Figure 11(1–6), along with the increase of arriving rate *λ*, the probability of state 0 decreases obviously. As the duration between state 0 and the next WBAN arrival declines, the Period Rate and Frequency Rate appear closer. On the contrary, the probabilities of large Coexisting-WBAN/Busy-channel Parameters increase obviously along with the arriving rate *λ*, which means that the interfering WBAN has a higher victim probability.

**Figure 12:**
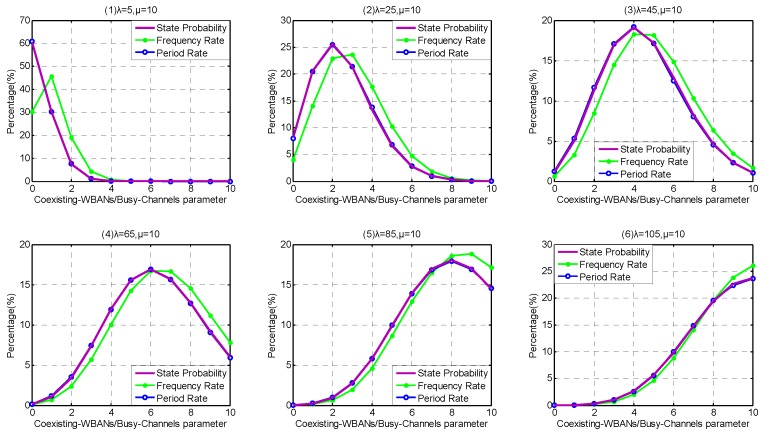
Coexisting-WBAN/Busy-channel Parameter probability distribution under 1-tolerated channel conflict model in Service-independent coexisting WBANs.

Secondly, because the coexistence capability in Service-independent coexisting WBANs is stronger than Service-dependent coexisting WBANs and can support higher arriving rate *λ*, we investigate the influence of arriving rate *λ* within the interval [5, 105], and the step is set to be 20, i.e., λ= {5, 25, 45, 65, 85, 105}. As shown in Figure 12, the similar phenomenon appears in Service- independent coexisting WBANs, that the Period Rate is closer to state probability distribution than Frequency Rate. When the Coexisting-WBAN Parameter is low, the Frequency Rate is lower than state probability and Period Rate. On the contrary, along with the increase of Coexisting-WBAN Parameter, the Frequency Rate becomes higher than state probability and Period Rate. This is because the leaving rate becomes higher and the state becomes instability when Coexisting-WBAN Parameter increases. From Figure 12(1–6), along with the increase of arriving rate *λ*, the distributions of state probability, Frequency Rate and Period Rate become more decentralized and the center shaft of them moves to the right, which also means that the interfering WBAN has a high probability to be conflicted.

Comparing Figure 11 and Figure 12, we obtain that Service-independent coexisting WBANs provides 7 times coexistence capability than Service-dependent coexisting WBANs if at most 25% Saturation Rate is permitted. As channel hopping operations waste energy and other network resources, Service-independent coexisting WBANs greatly outperforms the Service-dependent one on energy saving, latency, robustness and so on.

#### 6.1.2. *m*-Tolerated Channel Conflict Model

In *m*-tolerated channel conflict model, it is hard to provide theoretical solution for coexistence capability estimation. We present two approximated solutions based on our estimation methods. To simplify our description, the approximate method based on uniform distribution assumption is called Approximate Method I, and the estimation results of Approximate Method I is called Approximate Value I, abbreviated as Approx. Value I. Similarly, approximate method based on additive property of Possion-stream is called Approximate Method II, and the estimation results of Approximate Method II is called Approximate Value II, abbreviated as Approx. Value II.

We take coexistence capacity *m* = 3 for instance to investigate the Busy-channel Parameter probability distribution. Even the channel capacity is extended, we still investigate the same interval of parameter *λ* as 1-tolerated channel conflict model when considering queuing behavior, i.e., λ={1,4,7,10,13,16}. However, we extend the interval of the investigated parameter *λ* from 105 to 405, and the step is increased to 80 when considering non-queuing behavior, i.e., λ={5,85,165,245,325,405}.

**Figure 13:**
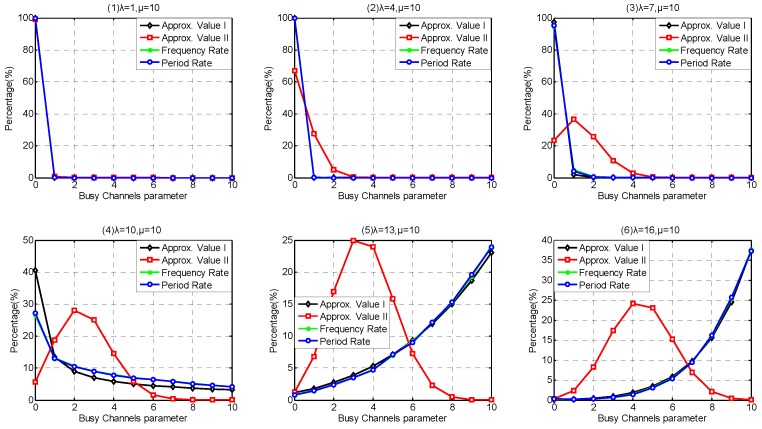
Busy-channel Parameter probability distribution under *m*-tolerated channel conflict model in Service-dependent coexisting WBANs.

**Figure 14:**
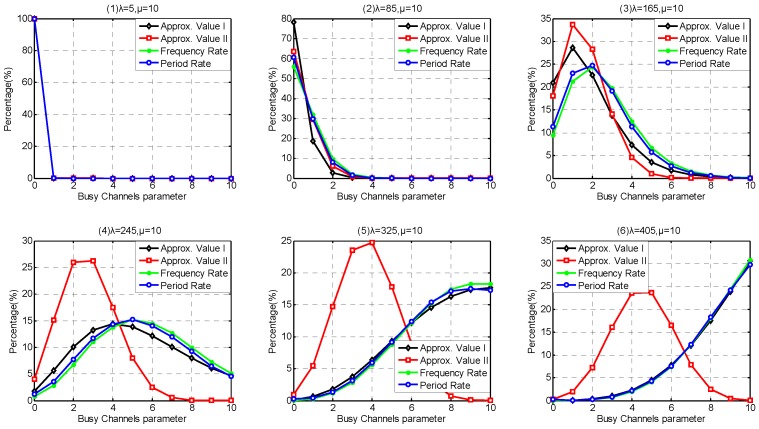
Busy-channel Parameter probability distribution under *m*-tolerated channel conflict model in Service-independent coexisting WBANs.

According to the simulation results discussed in Section 6.1.1, it is confirmed that Period Rate is suitable to indicate the state rate when investigate the coexistence capability under 1-tolerated channel conflict model. As shown in Figure 13 and Figure 14, the difference between Frequency Rate and Period Rate under 3-tolerated channel conflict model is much less than that under 1-tolerated channel conflict model, which is because the extension of channel coexistence capacity and the increase of arriving rate *λ* have weaken the effect of state 0, as well as the diversity of leaving rate $\mu_{k}$. As shown in Figure 13, Approximate Method I has a better probability distribution estimation accuracy than Approximate Method II in Service-dependent model. This is because when the arriving rate is low, we did not deal with the following operation after the channel is occupied, leading to coexistence capability underestimation. Along with the increase of arriving rate, the channels are easily occupied. More and more WBANs are refused due to the coexistence saturation, which is not respected in Approximate Method II. As shown in Figure 14, Approximate Method I still performs better than Approximate Method II in Service-independent coexisting WBANs except when λ≤85. This is because when the arriving rate is low, the channel occupied probability also performs low, and the effect of the following operation is neglectful.

Comparing Figure 11 and Figure 13, we notice that the extension of channel coexistence capacity barely benefits the coexistence capability of coexisting WABNs. While comparing Figure 12 and Figure 14, we obtain that limited channel coexistence capacity extension can substantially improve the coexistence capability and support more than 3 times larger interfering-WBAN arriving rate, which really benefits the performances of coexisting WBANs, such as energy saving, latency, robustness and so on.

### 6.2. Coexistence Capability Metrics Discussion

In this part, we investigate the high-level coexistence capability metrics including Coexisting Risk, Risk Variance, Saturation Rate, Interfering Period. We aim at investigating the influence of the channel coexistence capacity variation. From single WBAN supported to multiple WBANs supported, we increase the channel coexistence capacity from 1 to 6, i.e., m= {1, 2, 3, 4, 5, 6}, as analyzed in Section 2.2. Accordingly, we extend our *λ* simulation interval to 31 in Service-dependent coexisting WBANs and to 485 in Service-independent coexisting WBANs. As analyzed in Section 6.1, Period Rate is more suitable to indicate the state probability when investigating coexistence capability. So we employ Period Rate as our Simulation Value to evaluate our coexistence capability analysis and estimation.

**Figure 15:**
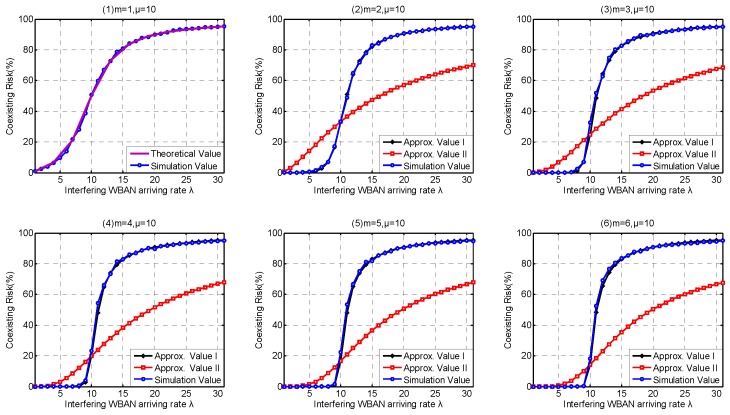
Coexisting Risk in Service-dependent coexisting WBANs.

**Figure 16:**
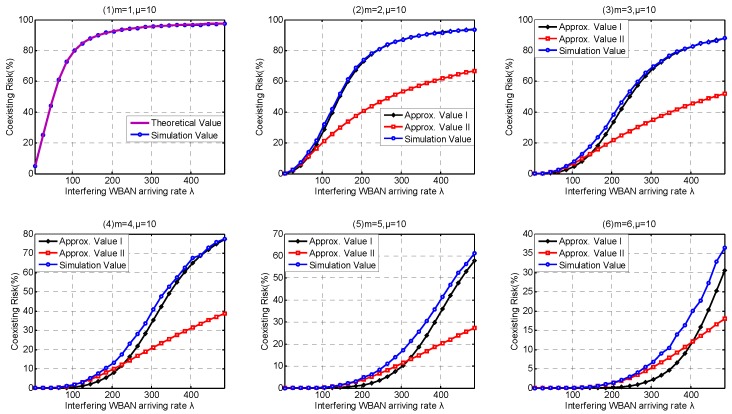
Coexisting Risk in Service-independent coexisting WBANs.

As shown in Figure 15(1), the theoretical value of Coexisting Risk shows a good agreement with the simulation value in Service-dependent coexisting WBANs. According to the subfigures (2–6) in Figure 15, Approximate Method I performs very close to the simulation value, and really beat Approximate Method II. Along with the increase of coexistence capacity and the decrease of *λ*, the approximate values of Coexisting Risk based on Approximate Method II are also improved. The correctness of our theory is also verified in Service-independent coexisting WBANs, refer to Figure 16(1). According to the subfigures (2–6) in Figure 16, Approximate Method I performs better than Approximate Method II when coexistence capacity is low and *λ* is large. This is because the channel is easier to be occupied in this scenario and the drawback of inaction with the following operations is exposed. Along with the increase of coexistence capacity and *λ*, the evaluation accuracy of Approximate Method I gets worse. This is because the rationality of equal probability distribution assumption for channel allocation is decreased, which is not reflected when only coexistence capacities 1 and 3 is investigated in Section 6.1. Fortunately, because of the negligible effect after channel occupied, Approximate Method II performs well with high coexistence capacity and low *λ*. According to the subfigures (2–6) in Figure 15, the benefits achieved from the extension of coexistence capacity are very limited in Service-dependent coexisting WBANs. On the contrary, the improvement of Coexisting Risk is extremely obvious in Service-independent coexisting WBANs. In consequence, numerous results are matching the similar problems in Section 6.1.2. As Coexisting Risk directly reflects the probability of a WBAN to be conflicted, the metric can also be employed to estimate resources consumption performance.

**Figure 17:**
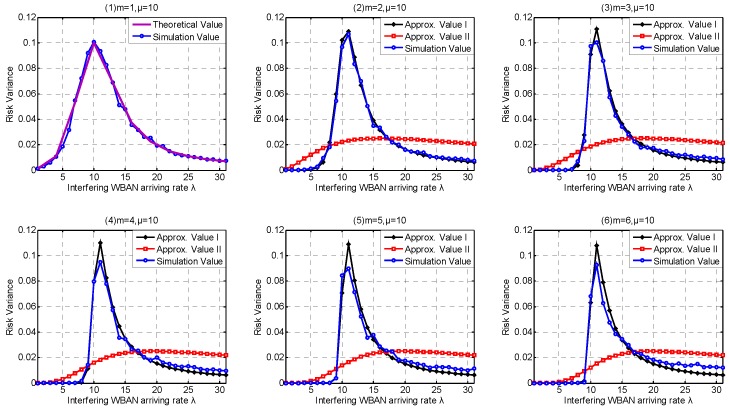
Risk Variance in Service-dependent coexisting WBANs.

**Figure 18:**
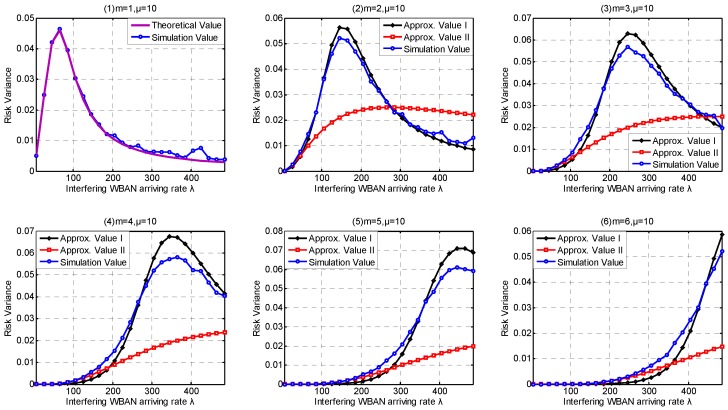
Risk Variance in Service-independent coexisting WBANs.

Accordingly, the correctness of our theory for Risk Variance estimation is also verified in both Service-independent and Service-dependent coexisting WBANs, referred to Figure 17(1) and Figure 18(1). Along with the increase of *λ*, the Risk Variance increases first, then decreases. This is because when *λ* is low, the average number of busy channels stay few with little probability transformation. As *λ* increases, the average number of busy channels increases and the range of transformation is extended until it increases to the maximum number of channels with little probability decrease. According to the Figure 17(2–6) and Figure 18(2–6) , Approximate Methods I and II show up similar properties on Risk Variance. What is noteworthy is that there are always two intersections between Approximate Methods I and II. The explanation we provide is that it is pure coincidence with especial parameter combination including *λ*, *μ*, number of channels, coexistence capacity. Interestingly, the two intersections must exist but there are no regulations to follow for ensuring if it is the most suitable parameter combination of an unknown application scenario.

**Figure 19:**
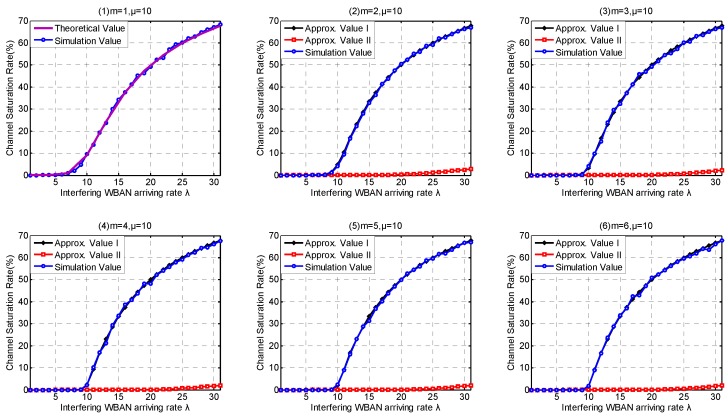
Channel Saturation Rate in Service-dependent coexisting WBANs.

**Figure 20:**
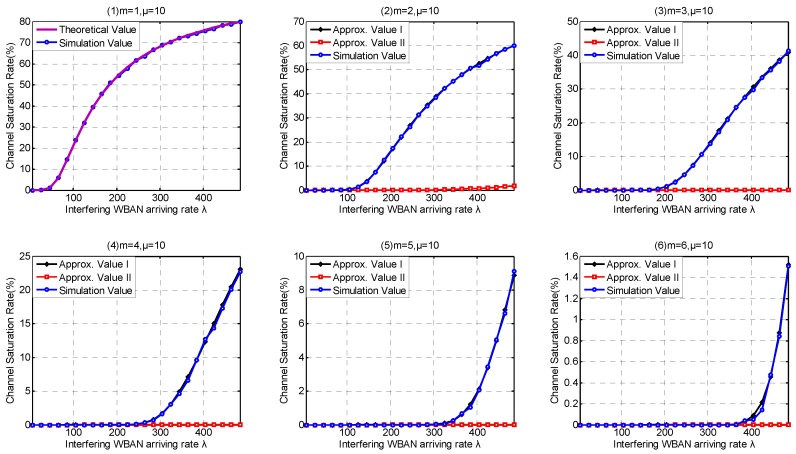
Channel Saturation Rate in Service-independent coexisting WBANs.

Similarly, Figure 19(1) and Figure 20(1) confirm that our theory for Channel Saturation Rate estimation is successful. As shown in Figure 19, the parameter coexistence capacity has little influences on the simulation result in Service-dependent coexisting WBANs, and the Channel Saturation Rate is mainly dependent on *λ*. On the contrary, both coexistence capacity and *λ* have obvious influences on Channel Saturation Rate, as shown in Figure 20. Comparing with Approximate Method I, Approximate Method II is totally defeated. Therefore, we don’t recommend employing Approximate Method II to estimate Saturation Rate. As the interfering WBANs have an inevitable coexistence conflict when coexisting WBANs are in Saturation State, the rejection of joining requires leads to terrible communication interruptions. Therefore, Channel Saturation Rate can also be employed to reflect the robustness.

**Figure 21:**
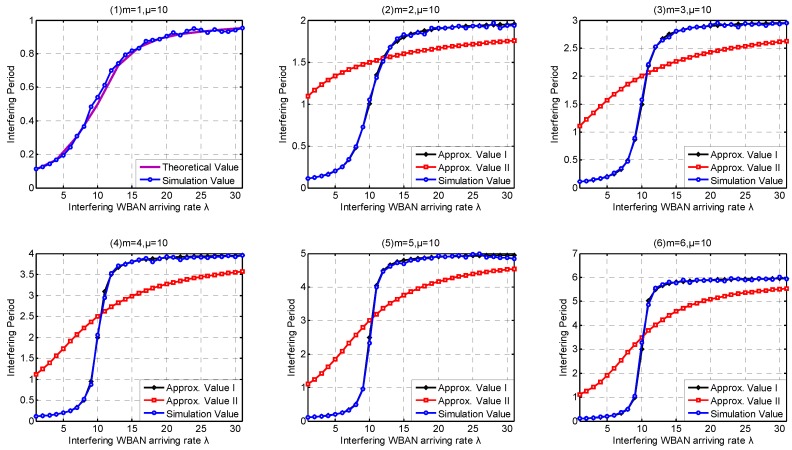
Interfering Period in Service-dependent model.

**Figure 22:**
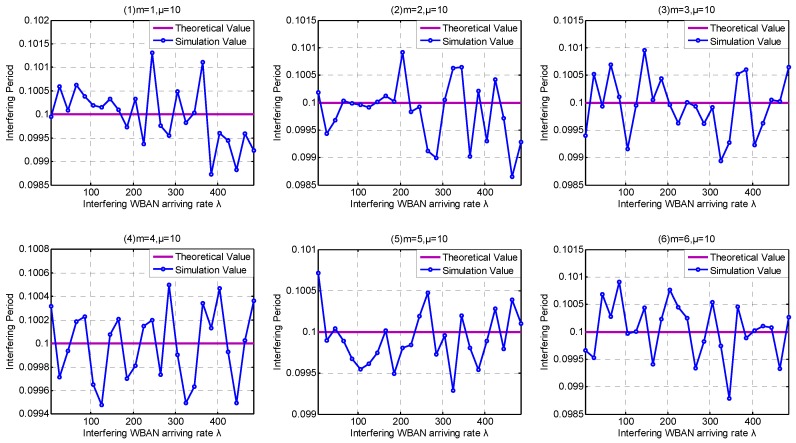
Interfering Period in Service-independent model.

As shown in Figure 21, Approximate Method I performs better than Approximate Method II on Interfering Period estimation. Interfering Period in Service-dependent coexisting WBANs increases along with the arriving rate *λ* until it reaches to m·n/μ, the upper bound of Interfering Period representing the residence period in saturation state. As analyzed in Section 5.2, we provided the theoretical value of Interfering Period in Service-independent coexisting WBANs. Because there is no queuing waiting time, the parameters we adopted have no effect on the Interfering Period except the leaving rate μ=10. As shown in Figure 22, the simulation values fluctuate around the simulation values.

Consequently, numerous simulation results show that our theoretical analysis is correct and our approximate estimation is so close to the real simulation value. Comparatively speaking, Approximate Method I has a great accuracy on all of the coexistence parameters and metrics involved in this paper. Approximate Method II performs better at Busy-channel Parameter estimation and Coexisting Risk estimation within special parameter interval in Service-independent coexisting WBANs.

### 6.3. Coexistence Performance Discussion

Since channel conflicts have a serious influence on the performance of coexisting WBAN, this part employs channel conflict times in each time unit to provide an intuitive performance evaluation of the enhanced channel hopping mechanism. Literature [12] has also employed the same evaluation indicator in their work.

According to our simulation results in Section 6.1 and Section 6.2, Service-dependent coexisting WBANs have a lower coexistence capability than Service-independent one. We investigate the coexistence performance with arriving rate *λ* = {9, 10, 11, 12} in the former and with arriving rate *λ* = {105, 205, 305, 405} in the latter. Furthermore, we implement the enhanced channel hopping mechanism with *m* = 1, *m* = 3 and *m* = 6, respectively. The situation *m* = 1 represents our enhanced channel hopping mechanism backoff to plain channel hopping mechanism.

**Figure 23:**
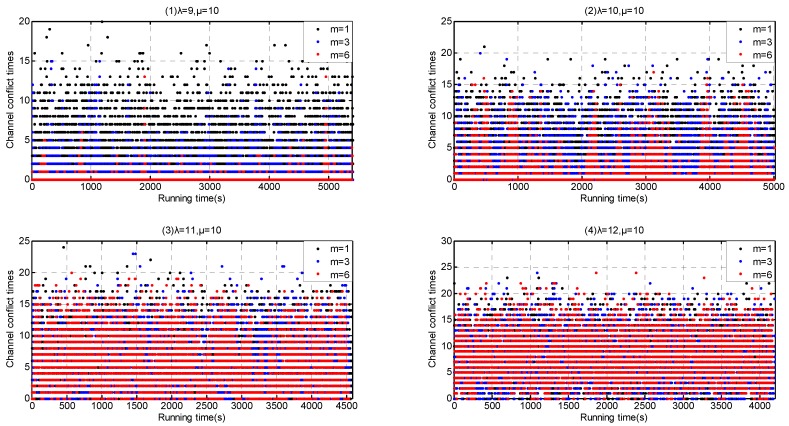
The times of channel conflicts in Service-dependent model.

**Figure 24:**
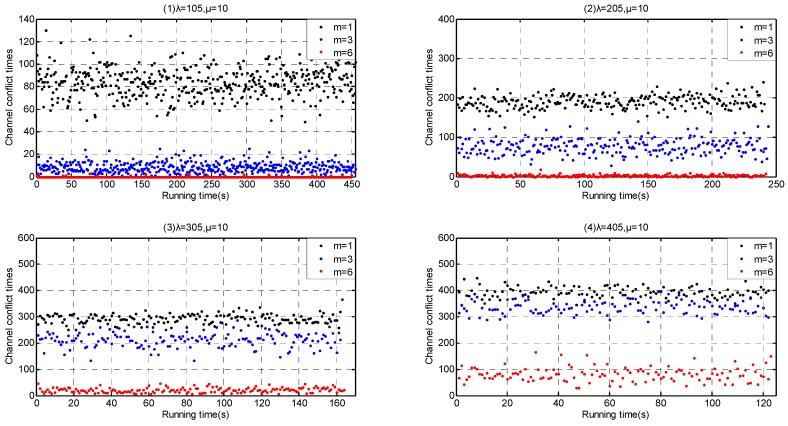
The times of channel conflicts in Service-independent model.

As shown in Figure 23, when λ≤ 10, the extension of channel coexistence capacity achieves an obvious channel conflict reduction in Service-dependent coexisting WBANs. While when λ> 10, the optimization fails. On the contrary, channel coexistence capacity extension mitigates the channel conflict greatly in Service-independent coexisting WBANs, as shown in Figure 24.

In consequence, our enhanced channel hopping mechanism outperforms the plain channel hopping.

## 7. Conclusions

This paper provided a comprehensive coexistence capability investigation for enhanced channel hopping mechanism in WBANs. Comparing with previous states of the art which focus on the algorithm designs of channel hopping sequences generation and the specific mechanism implementation, this paper aimed at extending the channel hopping mechanism to be more general by allowing multiple WBANs coexisted in the same channel. The channel resources were treated as logical blocks, which decoupled the implementation from integrating different channel-shared coexistence technologies. We provided comprehensive coexistence capability analysis and estimation approaches. The extensive simulation results showed that our coexistence parameters and metrics provided an advantage reflection for coexistence capability performances of channel hopping mechanisms. This paper provided a quantitative further guide for channel hopping exploitation. In the near future, we will optimize our approximate methods for more accurate estimation and lager parameter interval. Furthermore, complex scenarios with more arriving models and leaving models should also be taken into account in our further work.

## Figures and Tables

**Table 1:** The number of channel resources at each frequency.

Frequency Band (MHz)	Number of Channels (*Nch*)	Frequency Band (MHz)	Number of Channels (*Nch*)
402 to 405	10	950 to 958	16
420 to 450	12	2360 to 2400	39
863 to 870	14	2400 to 2483.5	79
902 to 928	60	–	–

**Table 2:** Recommended coexistence mechanisms.

Coexistence Mechanism	10 to 50 MHz HBC/EFC	402 to 405 MHz Band	868 MHz Band	902 to 928 MHz Band	2.4 GHz ISM Band	3.1 to 4.8 GHz and 6 to 10.6 GHz UWB Band
**Beacon shifting**	Not applicable	Not applicable given LBT restrictions	SD, D	SD, D	SD, D	D
**Channel hopping**	Not applicable	D	SD, D	SD, D	SD, D	S, SD, D for FM-UWB
**Active superframe interleaving**	S	None	None	S	S	S

**Table 3:** Parameter Table.

Parameters	Values	Parameters	Values
*λ*	2∼502	Number of channels	10
*μ*	10	Coexistence capacity	$\underline{1}$, 2, $\underline{3}$, 4, 5, 6
Simulation scale	50,000	–	–

## Appendix A.

### Appendix A.1. Abbreviations Table

**Table A1:** Abbreviations Table.

Abbreviations	Nomenclature	Abbreviations	Nomenclature
ACK	Acknowledgement	LFSR	Linear Feedback Shift Register
BDR	Beacon Delivery Ratio	MAC	Medium Access Control
CC	Coexistence Capacity	MBAA	Multiple-Beam Adaptive Array
CCA	Clear Channel Assessment	NB	Narrow Band
CSMA	Carrier Sense Multiple Access	PDR	Packet Drop Rate
D	Dynamic	PHY	Physical Layer
ECG	Electroardiograph	S	Static
EEG	Electroencephalogram	SD	Semi-dynamic
EMG	Electromyography	SIR	Signal to Interference Ratio
GPS	Global Position System	TDMA	Time Division Multiple Address
HBC	Human Body Channel	UWB	Ultra Wide Band
ISM	Industrial, Scientific and Medical	WBANs	Wireless Body Area Networks
LBT	Listen Before Talk		

### Appendix A.2. Symbols Table

**Table A2:** Symbols Table.

Article	Symbol	Article	Symbol
backoff delay threshold	$H_{switch}$	coexistence capacity	*m*
occupied channel statistics vector	$\bar{X}$	the number of WBANs in *i*-th channel	$\chi_{i}$
interfering WBAN channel index	*ι*	channel hopping sequence generator	G(*ι*,m,$\bar{X}$ )
coexisting state space	S	maximum index of coexisting state	N
coexisting-WBAN parameter	X	busy-channel parameter	Y
arriving rate	*λ*	serving rate	*μ*
the i-th state probability	$p_{i}$	Coexisting Risk	*R*
Risk Variance	*D*	Channel Utilization	U
*i*-channel busy rate	$p_{c}\left(i\right)$	average number of busy channels	$\bar{B}$
the probability of k busy channels	$p_{b}\left(k\right)$	saturation rate	$p_{s}$
expected value of queue length	L	Interfering Period	$T_{C}$
consumer residence time	$W_{s}$		
